# Reformation of the chondroitin sulfate glycocalyx enables progression of AR-independent prostate cancer

**DOI:** 10.1038/s41467-022-32530-7

**Published:** 2022-08-13

**Authors:** Nader Al-Nakouzi, Chris Kedong Wang, Htoo Zarni Oo, Irina Nelepcu, Nada Lallous, Charlotte B. Spliid, Nastaran Khazamipour, Joey Lo, Sarah Truong, Colin Collins, Desmond Hui, Shaghayegh Esfandnia, Hans Adomat, Thomas Mandel Clausen, Tobias Gustavsson, Swati Choudhary, Robert Dagil, Eva Corey, Yuzhuo Wang, Anne Chauchereau, Ladan Fazli, Jeffrey D. Esko, Ali Salanti, Peter S. Nelson, Martin E. Gleave, Mads Daugaard

**Affiliations:** 1 Department of Urologic Sciences, University of British Columbia, Vancouver, BC V5Z 1M9 Canada; 2 Vancouver Prostate Centre, Vancouver, BC V6H 3Z6 Canada; 3 Centre for Medical Parasitology at Department for Immunology and Microbiology, Faculty of Health and Medical Sciences, University of Copenhagen and Department of Infectious Disease, Copenhagen University Hospital, Copenhagen, Denmark; 4 Department of Cellular and Molecular Medicine, University of California San Diego, La Jolla, CA USA; 5 VAR2pharmaceuticals Ole Maaløes Vej 3, 2200 København, Denmark; 6 Department of Urology, University of Washington, Seattle, WA 98195 USA; 7 Prostate Cancer Group, INSERM UMR981, Gustave Roussy, University of Paris-Saclay, F-94805 Villejuif, France; 8 Fred Hutchinson Cancer Centre, Seattle, WA 98109-1024 USA

**Keywords:** Prostate cancer, Glycobiology, Molecular medicine

## Abstract

Lineage plasticity of prostate cancer is associated with resistance to androgen receptor (AR) pathway inhibition (ARPI) and supported by a reactive tumor microenvironment. Here we show that changes in chondroitin sulfate (CS), a major glycosaminoglycan component of the tumor cell glycocalyx and extracellular matrix, is AR-regulated and promotes the adaptive progression of castration-resistant prostate cancer (CRPC) after ARPI. AR directly represses transcription of the 4-*O*-sulfotransferase gene *CHST11* under basal androgen conditions, maintaining steady-state CS in prostate adenocarcinomas. When AR signaling is inhibited by ARPI or lost during progression to non-AR-driven CRPC as a consequence of lineage plasticity, CHST11 expression is unleashed, leading to elevated 4-*O*-sulfated chondroitin levels. Inhibition of the tumor cell CS glycocalyx delays CRPC progression, and impairs growth and motility of prostate cancer after ARPI. Thus, a reactive CS glycocalyx supports adaptive survival and treatment resistance after ARPI, representing a therapeutic opportunity in patients with advanced prostate cancer.

## Abstract (web summary)

Chondroitin sulfate (CS) is one of the most abundant glycosaminoglycans in prostate cancers. Here the authors show that inhibition of the androgen receptor pathway leads to the upregulation of CS, which promotes prostate cancer growth and metastasis.

## Introduction

Prostate cancer (PC) is a hormone-dependent disease driven by androgens. Androgen activates a nuclear receptor called the androgen receptor (AR) that controls gene expression via binding to AR response elements (AREs), distributed throughout the genome^1^. Depending on the molecular context and the localization of the AREs, AR can act as a transcriptional enhancer or repressor^2,3^. AR is essential for prostate adenocarcinoma cell viability and proliferation and androgen-deprivation therapy (ADT) is therefore used as a first-line treatment to control tumor growth^4,5^. While ADT is usually initially effective, prostate cancer is characterized by the predictable emergence of resistance and progression towards castration-resistant prostate cancer (CRPC)^6^. This clinical scenario has prompted development of second-line AR signaling inhibitors, such as enzalutamide and abiraterone, that have extended overall survival of CRPC patients^7–10^, and are now approved in combination with ADT in first-line metastatic prostate cancer^11–14^. Many studies indicate that androgen-receptor pathway inhibition (ARPI) induces a cascade of changes in DNA structure, gene expression, and selective translation of proteins that regulate signaling networks to provide survival of sub-populations of prostate cancer cells^15^. Certain prostate cancer subtypes with loss of p53, RB, BRCA, or PTEN are Darwinian fit and better primed to survive, adapt, and progress after ARPI. Under the selective pressures of ARPI, genomic alterations are being selected that most commonly support reactivation of AR signaling^15,16^. However, about 20% of CRPC manifests stem and developmental pathways that are activated to support emergence of alternate lineage phenotypes adapted to progress in absence of AR activity^17^. Therefore, identification of survival mechanisms facilitating adaptation of prostate cancer to an AR-indifferent state is an emerging need to prolong responses to ARPI.

The glycocalyx is a pericellular matrix of multifunctional glycoproteins and glycosaminoglycans (GAG) coating all living cells. The tumor cell glycocalyx is often bulkier than that of normal cells and is thought to facilitate cell–cell biochemical communication and structural support in the tumor^18–24^. Alterations in expression and composition of GAGs and proteoglycans have been sporadically described in prostate cancer for more than four decades^25–36^. In this work, we investigated the role and regulation of the tumor cell glycocalyx in PC progression. We show that *CHST11* is an AR-repressed gene and that CSA is an essential component of the prostate tumor glycocalyx that supports progression of AR-indifferent CRPC.

## Results

### Castration alters expression of glycosaminoglycan enzymes in a PDX model of prostate cancer progression

**Fig. 1:**
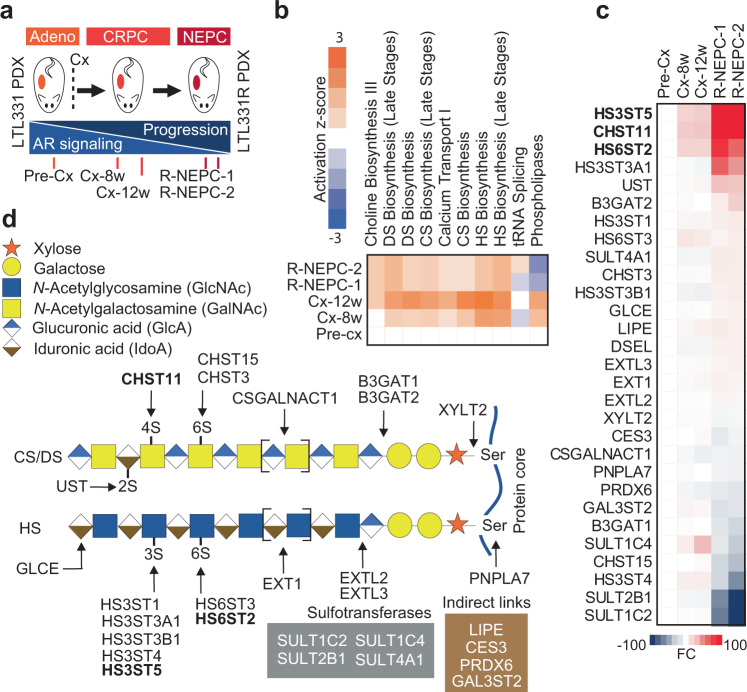
Castration alters expression of glycosaminoglycan enzymes in a patient-derived xenograft model of prostate cancer progression. **a** Overview of the LTL331 PDX model and schematic depicting time points at which tumors were collected. **b** IPA-ingenuity analysis on RNA-Seq data from LTL331 transdifferentiation PDX model showing the top upregulated metabolic pathways. **c** Fold change heat map of IPA gene expression analysis showing the modified glycosaminoglycan genes. **d** Function of genes identified in **c** in GAGs synthesis pathways. arrows indicate the site of activity of the listed enzymes. Gray box includes other relevant sulfotransferases brown box include identified genes with indirect link to GAGs synthesis pathway. Source data are provided as a Source Data file.

The patient-derived xenograft (PDX) trans-differentiation model (LTL331/LTL331R) is a typical hormone-naïve AR/PSA-positive adenocarcinoma that upon castration initially regresses, but eventually relapses as terminally-differentiated AR-negative neuroendocrine prostate cancer (NEPC) (LTL331R) (Fig. 1a), adequately reflecting the clinical scenario in humans following ARPI^37,38^. We performed transcriptomic analysis on LTL331/LTL331R tumors from different disease stages pre- and post-castration (Fig. 1a) (https://www.ncbi.nlm.nih.gov/geo/query/acc.cgi?acc=GSE59986), followed by an ingenuity pathway analysis of glycosylation-associated pathway alterations. The analysis revealed that GAG-related pathways, in particular those leading to chondroitin sulfate and heparan sulfate synthesis, were upregulated after murine castration and in the NEPC relapse disease stage (Fig. 1b). In total, 29 enzymes involved in initiation, elongation, and sulfation of GAG chains were significantly modified with fold changes of expression ranging from 2 to 100 (Fig. 1c, d). The top three genes upregulated after castration as compared to adenocarcinomas were the heparan sulfate sulfotransferases *HS3ST5* and *HS6ST2*, and the chondroitin sulfate sulfotransferase *CHST11* (Fig. 1c). These data indicate that glycosaminoglycan enzyme is affected by ARPI in prostate cancer and may be associated with progression from adenocarcinomas to CRPC and NEPC disease stages.

### Prostate tumors with loss of AR activity display elevated CHST11 and chondroitin sulfate levels

**Fig. 2:**
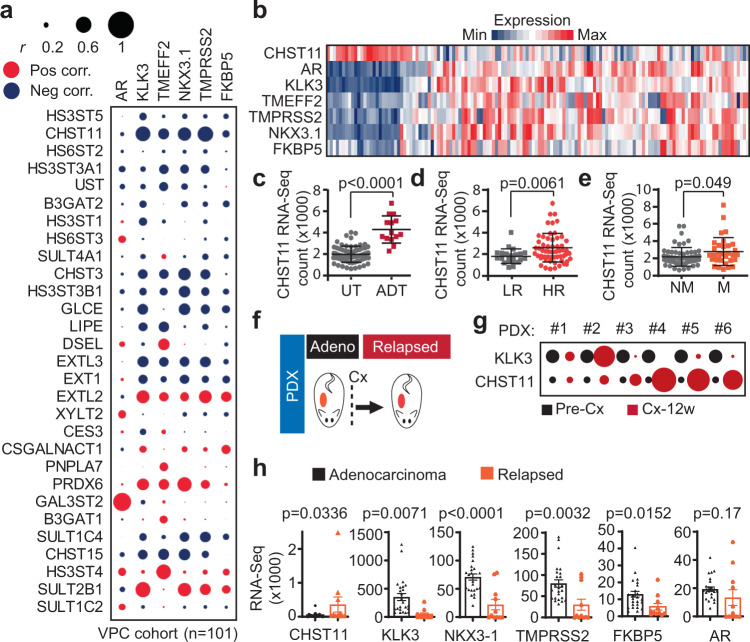
Patient tumors with loss of AR activity display elevated CHST11 and chondroitin sulfate levels. **a** Correlation analysis of mRNA levels of GAGs biosynthesis genes identified in Fig. 1c and AR regulated genes (*KLK3, TMEFF2, NKX3.1, TMPRSS2, FKBP5*). Circle size represents Pearson correlation coefficient absolute values. Blue, negative correlation; Red, positive correlation, VPC cohort (*n* = 101)^39^. **b** Heatmap of gene expression in CRPC patients; (UW cohort; *n* = 138) [https://www.ncbi.nlm.nih.gov/geo/query/acc.cgi?acc=GSE147250] (GSE147250). **c** *CHST11* gene expression in untreated (*n* = 69) and ADT treated PC patients (*n* = 14) (mean ± SD; two-tailed Mann-Whitney test); **d** low-risk (LR, *n* = 22) and high-risk (HR, *n* = 57) (mean ± SD; two-tailed Mann-Whitney test); **e** patients with metastases (M; *n* = 33) and without metastases (NM; *n* = 58) (mean ± SD; two-tailed Mann-Whitney test). (VPC cohort). **f** Schematic protocol of PDXs tumor generation for RNA-Seq analysis used in **g** and **h**. **g** *CHST11* and KLK3 gene expression in prostate cancer (PC) PDXs before (Pre-Cx) and 12 weeks after castration (Cx-12w). Circle size represents fold change of normalized RNA-Seq counts of Cx-12w compared to Pre-Cx in each PDX. **h** RNA-Seq gene expression of *CHST11* and AR regulated genes in Adeno (*n* = 26) and relapsed (*n* = 11) PC PDXs. (mean ± SEM; two-tailed t-test). Source data are provided as a Source Data file.

We next validated the results from the PDX discovery model in prostate cancer patients. We first determined the relationship between the identified 29 enzyme-coding genes (Fig. 1c) and androgen signaling in a clinical RNA-Seq dataset of 101 prostate cancer patient tumors at different disease stages (VPC cohort)^39^. We correlated expression of the 29 genes with an expression of five validated AR-regulated genes *KLK3*, *NKX3.1*^40^, *TMPRSS2*^41–43^, *FKBP5*^44^, and *TMEFF2*^45^ as readouts for AR signaling activity. Of the top-three genes (i.e., HS3ST5, *HS6ST2*, and *CHST11*), only *CHST11* showed a consistent negative correlation with all five AR-regulated genes (Fig. 2a and Supplementary Fig. 1a). The result was corroborated in a second validation cohort of *n* > 196 mixed stage patients (IST cohort) (Supplementary Fig. 1b), supporting an inverse relationship between *CHST11* expression and AR signaling in prostate cancer. To further validate that idea, we analyzed a third cohort comprised of 138 CRPC patients (University of Washington; UW cohort) (https://www.ncbi.nlm.nih.gov/geo/query/acc.cgi?acc=GSE147250). Here, *CHST11* expression alone was able to segregate patients based on loss of AR activity (Fig. 2b). We next analyzed a cohort of prostate cancer patients pre- and post-treatment with ADT and with respect to risk and metastatic disease. Here, *CHST11* expression was elevated in patients treated with ADT (Fig. 2c), in high-risk tumors (Fig. 2d), and in patients with metastatic disease (Fig. 2e).

Some patients that progress with CRPC after ADT have tumors with proficient AR signaling despite castrate levels of serum androgens, while others progress with tumors that are independent of AR pathway activity^46–49^. We next assessed whether *CHST11* expression could be directly linked to AR activity in PDX tumors matched and un-matched pre- and post-castration (Fig. 2f). *CHST11* expression levels were increased in relapsed matched PDX tumors only when AR activity was impaired, as indicated by loss of PSA (KLK3) expression (Fig. 2g). Moreover, in a larger unmatched PDX cohort of naïve (*n* = 26) and ADT-relapsed (*n* = 11) CRPC tumors, *CHST11* up-regulation was associated with decreased levels of the AR-regulated genes (i.e., KLK3, *NKX3-1*, *TMPRSS2*, and *FKBP5*), while there was no association with expression of AR (Fig. 2h). Combined, these data show that elevated *CHST11* expression inversely correlates with AR signaling activity in prostate tumors.

### CHST11 is an AR repressed gene

**Fig. 3:**
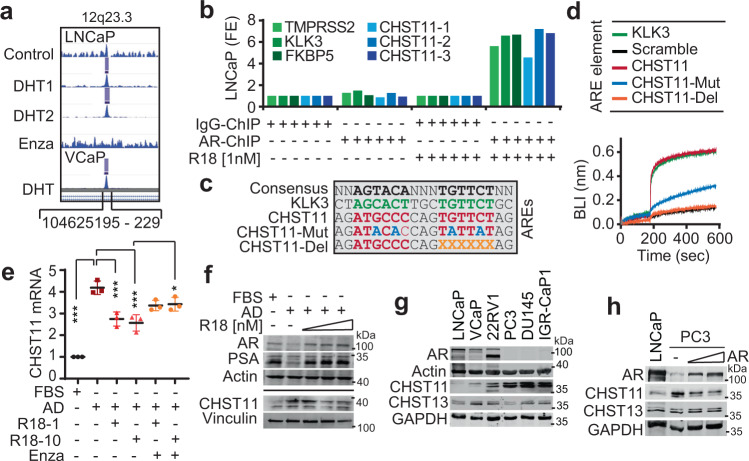
CHST11 is an AR repressed gene. **a** ChIP-seq AR binding intensity within indicated *CHST11* genomic region in LNCaP and VCaP cells (Cistrome.com; WashU Epigenome Browser) (^50^, ^51^). **b** qPCR analysis using three sets of CHST11 primers designed around the Chr 12:104625188 location, after CHIP of AR in LNCaP cells in CSS media in presence or absence of R1881. TMPRSS2, FKBP5, and PSA validated primers around the ARE were used as positive controls. **c** Identified CHST11 ARE, mutated and deleted sequences. **d** BLI assay where biotinylated AR-DNA binding domain was immobilized sensor and incubated in presence of CHST11 ARE sequences listed in **c**, KLK3-ARE sequence used as positive control. Representative data of 3 independent experiments. **e** Levels of *CHST11* transcripts by q-PCR in LNCaP cells after 72 h of androgen deprivation (AD) condition and 48 h of treatment with R1881 and/or Enzalutamide normalized to FBS condition. *n* = 3 independent biological samples; mean ± SD; FBS vs. AD *p* < 0.0001; AD vs. AD + R1881 [1 nM] *p* = 0.0006; AD vs. AD + R1881 [10 nM] *p* = 0.0002; AD + R1881 [10 nM] vs. AD + R1881 [10 nM] +Enzalutamide *p* = 0.0314. One-way ANOVA followed by Tukey correction *p < 0.05; ***p < 0.001). **f** Protein levels of PSA, CHST11, and AR in LNCaP cells in FBS or AD condition ± R1881 (1, 5, 10 nM); representative of three independent experiments. **g** Protein levels of CHST11 and CHST13 in AR + and AR- PC cell lines. The CHST11 antibody detected additional uncharacterized bands at ~30 kDa (see Source data file); representative of four independent experiments. **h** CHST11 and CHST13 protein levels in PC3 after AR ectopic expression for 72 h. LNCaP lysate was used as reference. Experiment performed in duplicate. Source data are provided as a Source Data file.

The upregulation of *CHST11* expression in patient tumors after ARPI hints that the *CHST11* gene might be repressed by AR. To test this, we first assessed the *CHST11* gene signature in an AR chromatin-IP (ChIP) sequencing dataset of LNCaP cells cultured with or without androgens and/or treatment with the clinical-grade AR inhibitor, enzalutamide^50^. The analysis identified a strong peak in the first intron of the *CHST11* gene located at 12q23.3 (104625195-229) in LNCaP cells treated with Dihydrotestosterone (DHT) and blocked by enzalutamide (Fig. 3a). The peak found in LNCaP cells was also observed in androgen-sensitive VCaP cells cultured in the presence of DHT (Fig. 3a), indicating that AR directly binds the *CHST11* gene. We next confirmed the binding of AR to the *CHST11* gene by ChIP-PCR in LNCaP cells using three nonoverlapping primer sets flanking the 104625195-229 region of 12q23.3. The three primer sets were able to amplify the genetic region in *CHST11* under androgen-proficient conditions only, and the amplification pattern was similar to that of three known AR target genes *TMPRSS2*, *KLK3*, and *FKBP5* (Fig. 3b). In the center of the identified peak and in the ChIP-PCR amplified *CHST11* sequences, we identified a putative ARE with strong consensus to the ARE found in the *KLK3* gene (Fig. 3c). To test whether the putative ARE in *CHST11* was functional, we first performed a bio-layer interferometry (BLI) analysis measuring the physical interaction between recombinant AR protein and wildtype, mutated, or deleted versions of the putative ARE sequence (Fig. 3c, d). AR bound the *CHST11* ARE with the same kinetics as the PSA ARE, but failed to bind mutated or partially deleted forms of the *CHST11* ARE (Fig. 3d). Also, electrophoretic mobility shift assay (EMSA) using purified AR DNA binding domain (DBD) revealed a dose-dependent interaction between recombinant AR-DBD protein and a labeled *CHST11*-ARE sequence similar to that observed with a labeled PSA-ARE sequence (Supplementary Fig. S2a). Moreover, in a competitive EMSA setting, the unlabeled version of the *CHST11*-ARE probe reduced the binding between the AR-DBD protein and the TMPRSS2-ARE sequence (Supplementary Fig. S2b), confirming a physical interaction between the *CHST11*-ARE and AR. We next investigated whether the *CHST11-*ARE was functional in prostate cancer cells. Upregulation of the CHST11 mRNA transcript was induced by AD in androgen-sensitive LNCaP cells that could be reverted by methyltrienolone (R1881) addition, a synthetic androgen agonist, and counter-rescued by addition of enzalutamide (Fig. 3e). Moreover, LNCaP-derived AR-low neuroendocrine-like 42D cells displayed elevated CHST11 levels while AR-positive LNCaP-derived V16D CRPC cells expressed CHST11 to the same levels as wild-type LNCaP cells (Supplementary Fig. S2c). We then examined CHST11 and PSA expression levels after manipulation of AR activity in androgen-sensitive LNCaP cells. AD conditions increased CHST11 and decreased PSA protein levels in LNCaP cells which could be reversed by addition of R1881 (Fig. 3f and Supplementary Fig. S2d). We next determined CHST11 protein and mRNA levels in AR-negative cell lines (PC3, DU145, IGR-CaP1) as compared to AR-positive cell lines (LNCaP, VCaP, 22RV1). The AR-negative cells had consistently higher CHST11 mRNA and protein expression than the AR-positive cells (Fig. 3g and Supplementary Fig. S2e, f). Finally, we reconstituted AR expression in AR-negative PC-3 cells and evaluated the impact on CHST11 expression. AR-reconstituted PC-3 cells had reduced CHST11 expression to levels that were comparable to AR-positive LNCaP cells (Fig. 3h). Combined, these data show that *CHST11* is an AR-repressed gene in prostate cancer.

### Expression of chondroitin sulfate A is controlled by AR via CHST11

**Fig. 4:**
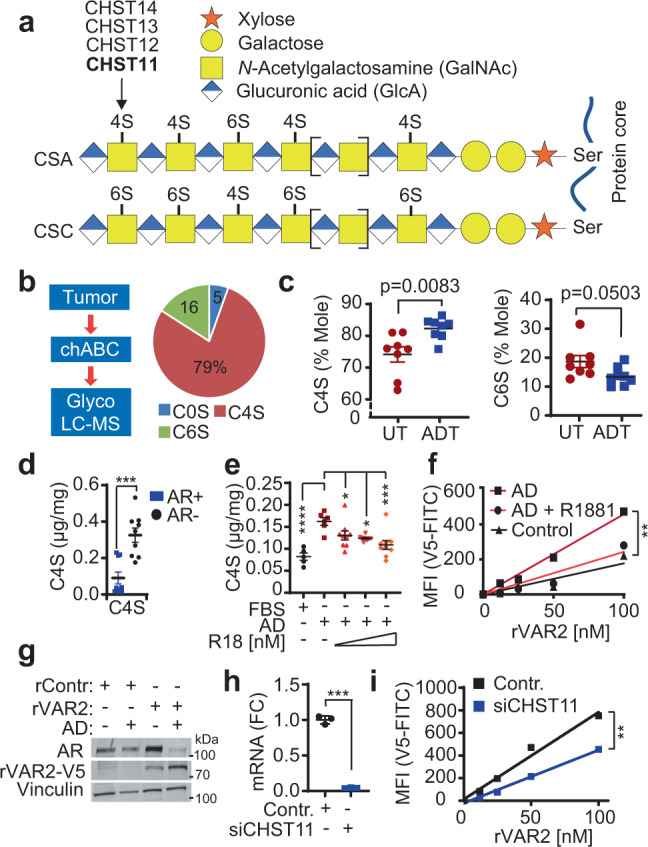
Expression of chondroitin sulfate A is controlled by AR in prostate cancer. **a** Schematic of CS chains, their sulfations sites C4S and C6S. **b** CS was purified from paraffin fixed PC patients’ samples using chondroitinase ABC enzyme (Chase) and subjected to LC-MS analysis. The C0S, C4S, and C6S mole percentages were calculated as the percentage of the total moles of disaccharides (C0S + C4S + C6S). Data are shown as the average percentage of moles of 16 PC patients. **c** Quantification of C4S and C6S of tissues from androgen deprivation therapy- treated (ADT, *n* = 8) and untreated (UT, *n* = 8) high-risk PC patients using LC-MS; mean ± SEM; Two tailed *t* test . **d** LC-MS C4S levels in AR (+) cell lines (LNCaP, VCaP, 22RV1) and AR (−) cell lines (PC3, Du145, IGR-CaP1). *n* = 9 samples (three cell lines and three biologically independent replicates); mean ± SEM; *p* = 0.0003 by two-tailed *t* test. **e** LC-MS C4S quantification in LNCaP cells after 72 h of AD condition and 48 h of treatment with 1, 5, or 10 nM of R1881. AD *n* = 6; FBS *n* = 5, *p* < 0.0001; AD + R18[1 nM] *n* = 9, *p* = 0.05; AD + R18[5 nM] *n* = 9, *p* = 0.014; AD + R18[10 nM] *n* = 9 biologically independent samples, *p* = 0.0006 by one-way ANOVA followed by Dunnett correction; data represented as mean ± SEM. **f** CSA expression levels in LNCaP cells in ambient conditions and AD conditions (7 days) determined by flow cytometry using rVAR2 lectin; linear regression followed by Ancova test; *p* = 0.0272, *F* = 5.529, DFn = 2, DFd = 9. **g** CSA and AR expression levels in LNCaP cells in ambient and AD conditions (7 days) determined by western blotting using rVAR2-V5 lectin and V5 antibody. **h** *CHST11* mRNA levels 48 h after *CHST11* knockdown. Data are shown as fold change (FC) mean ± SD; *n* = 3 biological replicates. **i** CSA expression levels in PC3 cells 48 h after siCHST11 determined by flow cytometry using rVAR2 lectin and anti-V5 antibody. linear regression followed by Ancova test; *p* = 0.0448, *F* = 6.387, DFn = 1, DFd = 6. Representative data of three independent experiments. *p < 0.05; **p < 0.01; ***p < 0.001; ****p < 0.0001. Source data are provided as a Source Data file.

CS chains can carry different sulfations on their sugar residues. If a CS chain is predominantly sulfated on carbon-4 (C4S) of the GalNAc residues, it is referred to as CS type A (CSA). If a CS chain is mostly sulfated on GalNAc carbon-6 (C6S), it is referred to as CS type C (CSC). While seen less frequently, CS chains can sometimes be C4S/C6S double-sulfated (CSE), or they can carry a carbon-2 sulfation of the GlcA residue in combination with a GalNAc C6S (CSD). CHST11 is a 4-*O*-sulfotransferase that catalyzes C4S sulfations on CS chains and promotes the synthesis of CSA-type CS^52^ (Fig. 4a). CHST12, CHST13, and CHST14 can also mediate C4S sulfations under some circumstances^53,54^, however, these enzymes did not respond to castration in the prostate cancer progression PDX model (Supplementary Fig. S3a). Moreover, expression of CHST13, the closest paralogue to CHST11, was close to absent in primary patient tumors (Supplementary Fig. S3b) and unaffected by ARPI in both PDX models and patients (Supplementary Fig. S3c, d).

To determine the baseline levels of CSA and CSC in prostate cancer, we analyzed sulfation composition of GlcA-GalNAc disaccharides in 16 PDX prostate tumors by LC-MS. The analysis showed that these tumors had an average CS composition of 5% C0S (unsulfated), 16% C6S, and 79% C4S disaccharides (Fig. 4b and Supplementary Fig. S4a), indicating that the majority of CS chains in prostate cancer is CSA type. We next investigated if CSA levels were changing in prostate tumors after ARPI. We analyzed the C4S content of untreated (*n* = 8) or ADT-treated (*n* = 8) tumors from prostate cancer patients by LC-MS. Neoadjuvant-ADT treated tumors with high *CHST11* (Fig. 2c, h) presented with elevated levels of C4S, and reduced levels of C6S, as compared to treatment-naïve tumors (Fig. 4c). In agreement, AR-negative cells (PC3, DU145, IGR-CaP1) had higher average levels of C4S disaccharides as compared to AR-positive cells (LNCaP, VCaP, 22RV1) (Fig. 4d). C4S disaccharides were increased in LNCaP cells subjected to androgen deprivation, which could be reverted by addition of R1881 (Fig. 4e). Similarly, CS56 staining increased after androgen deprivation and was rescued by R1181 addition (Supplementary Fig. S4b). The increase in C4S disaccharides after androgen deprivation suggests that CSA is increased. Indeed, androgen-deprived LNCaP cells exhibited increased binding to the CSA-specific rVAR2 lectin, which could be reverted by addition of R1881 (Fig. 4f). The increase of CSA chains on LNCaP cells after androgen deprivation was confirmed by immunoblotting of cell-bound rVAR2 (Fig. 4g). In order to rule out that the observed CSA changes are due to CSPGs core proteins being AR regulated, we examined the expression levels of major CSPGS in different PC cell lines from the dataset published in ref. 55. The analysis showed that while AR expression and activity correlated positively with TMEFF2 and negatively with CD44, it did not correlate with the ten remaining CSPGs known to be able to carry CSA modifications (Supplementary Fig. S4c). This result was further confirmed on a selected panel of cell lines and CSPGs by qRT-PCR and western blotting (Supplementary Fig. S4d, e). Together, these data indicate that CSA regulation by AR is independent of the CSPG core proteins. We next investigated whether CHST11 indeed was responsible for CSA synthesis in prostate cancer. Knockdown of CHST11 with siRNAs induced cell death in both AR-proficient LNCaP (Supplementary Fig. S5a–d) and AR-negative PC-3 (Supplementary Fig. S5e, f) cells after 72–96 h, indicating that basal levels of CSA are required for survival of PC cells independent of AR signaling. However, pre-cell death assessment of CSA expression at 48 h after *CHST11* knockdown (Fig. 4h), showed a significant reduction of CSA chains in PC-3 cells (Fig. 4i), supporting the idea that CHST11 is driving CSA expression in prostate cancer. Combined, these data show that CSA constitutes the majority of CS in prostate cancer and is regulated by AR via CHST11.

### Prostate cancer depends on high chondroitin sulfate levels after ARPI

**Fig. 5:**
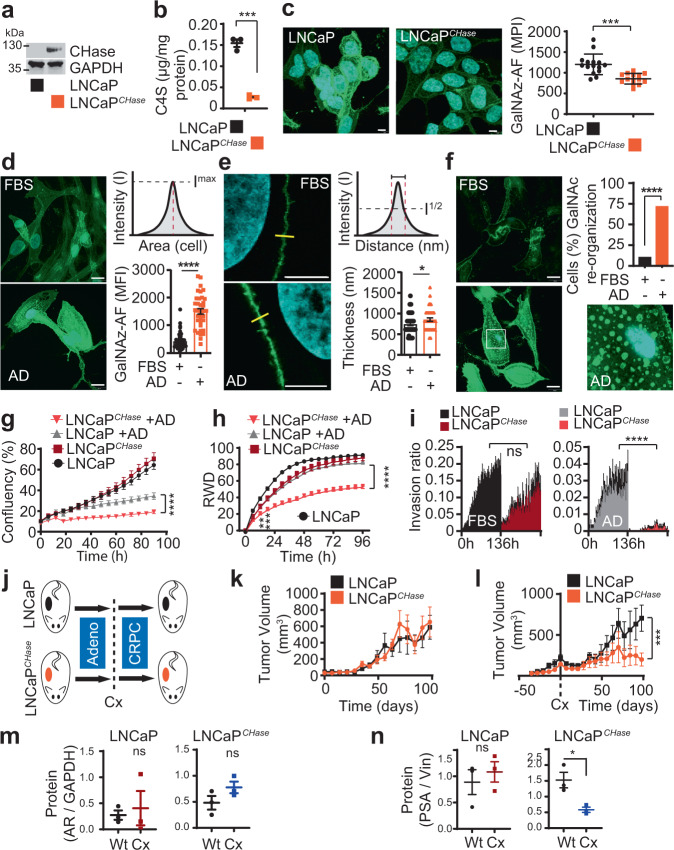
Prostate cancer becomes dependent on high chondroitin sulfate levels after ARPI. **a** Expression of CHase in LNCaP^CHase^ and parental cell lines by western blotting. **b** C4S quantification in LNCaP and LNCaP^CHase^ cell lines using LC-MS. C4S content normalized to protein content and shown as mean ± SEM; *n* = 3 independent samples; *p* = 0.0002 by two-tailed *t* test. **c** CS quantification using Gal-NAz incorporation followed by Cu click of alkyne-488 on LNCaP and LNCaP^CHase^ cells; data shown as mean of maximum pixel intensity (MPI) ± SD. *n* = 15 fields of view; *p* < 0.0001 by two-tailed *t* test. **d** Levels of GalNAc in LNCaP cells glycocalyx determined in AD and normal conditions (FBS) by immunofluorescence using GalNAz followed by Cu click of alkyne-488 (green). DAPI staining (blue) was used to identify the nucleus. Scale bar is 10 μm. Data are shown as mean ± SEM; *p* < 0.0001 by two-tailed *t* test. (FBS, *n* = 45 ROI; AD, *n* = 35 ROI). **e** Thickness of GalNAc positive cell membranes. Data are shown as mean ± SEM; (FBS *n* = 48; AD *n* = 39); *p* = 0.046 by two tailed *t* test. **f** Glycocalyx re-organization in AD condition. Data are expressed as percentage of cells with GalNAz spots at cell basement (*n* = 11 fields of view); *p* < 0.0001 by Chi-squared test. **g** Growth (IncuCyte) of LNCaP and LNCaP^CHase^ cells in FBS or AD conditions at different time points. Data are shown as mean ± SD; FBS *n* = 16; AD, *n* = 6 independent samples; *p* < 0.0001 after 24 h; 2 two-way ANOVA followed by Tukey’s correction. **h** Relative wound density (RWD) measurements of LNCaP and LNCaP^CHase^ cells in FBS or AD conditions. Data are shown as mean ± SD; *n* = 4 independent samples; *p* < 0.0001 after 24 h; 2 two-way ANOVA followed by Tukey’s correction. **i** Invasion capacity (IncuCyte; Chemotaxis assay) of LNCaP^CHase^ and parental cell lines in FBS and AD conditions for 136 h. The recipient well-contained media + 10% FBS as chemoattractant. Results are expressed as phase object count normalized to initial Top value (bottom) and shown as mean ± SEM. *n* = 4 independent samples; *p* < 0.0001 after 24 h; 2 two-way ANOVA followed by Tukey’s correction. **j** Protocol schematic of LNCaP and LNCaP^CHase^ xenografts growth in ambient and castration conditions. **k** Tumor growth of LNCaP and LNCaP^CHase^ xenograft without or **l** after castration. Data are shown as mean of tumor size ± SEM; *n* = 5 animals; *p* = 0.031 by Mann-Whitney test on slopes after castration from nadir to endpoint. **m** Western blot intensity quantification of AR levels from tumors in Figs. 5k and 5l; data represents AR to GAPDH intensity ratio and shown as mean ± SEM; *n* = 3 biologically independent tumor samples. **n** Western blot intensity quantification of PSA levels from tumors in Fig. 5k and l; data represent PSA to vinculin intensity ratio and shown as mean ± SEM; *n* = 3 biologically independent tumor samples; *p* = 0.022 by two tailed *t* test. ns: non-significant, *p < 0.05; **p < 0.01; ***P < 0.001; ****P < 0.0001. Source data are provided as a Source Data file.

We next investigated the functional role of CSA in CRPC. We engineered LNCaP cells to express chondroitinase ABC (LNCaP^CHase^), which is an enzyme that degrades CS after it is presented on proteoglycans in the cell plasma membrane (Fig. 5a and Supplementary Fig. S6a)^56,57^. LNCaP^CHase^ cells did not have complete depletion of C4S but displayed ~80% lower C4S levels as compared to wildtype cells (Fig. 5b). We next assessed morphological and functional consequences of CS reduction in prostate cancer cells after ARPI. CS is expressed as modifications to proteoglycans (CSPGs) in cell plasma membranes as part of the cellular glycocalyx^58–61^. While a single GalNAc residue is found in the stem of all *O*-glycans, a large portion of GalNAc residues is found in CS, where it represents ~50% of the GAG chain (Fig. 4a). The GalNAc mimic N-azidoacetylgalactosamine (Gal-NAz) contains an azide group in the sugar ring that allows for conjugation of fluorescent probes, providing a tool for monitoring the GalNAc-containing glycocalyx in real time^62^. As CS contains high levels of GalNAc (Fig. 4a–f), we first tested whether incorporation of Gal-NAz could visualize changes in the CS glycocalyx using LNCaP and LNCaP^CHase^ cells. Indeed, LNCaP^CHase^ cells displayed a significant reduction in Gal-NAz signal as compared to wildtype cells (Fig. 5c, Supplementary Fig. S6b, c), reflecting that a large and adequate portion of GalNAc residues is CS incorporated in prostate cancer.

With a tool to monitor the CS glycocalyx based on GalNAc tracing, we next assessed the impact of ARPI on the GalNAc-containing glycocalyx in LNCaP cells using real-time Gal-NAz labeling. We cultured LNCaP cells in AD conditions for 7 days where Gal-NAz was added on day 5 and subsequently Cu-click live-conjugated with Alexa Fluor-488-alkyne to visualize real-time androgen-dependent changes in the GalNAc-containing glycocalyx. Compared to LNCaP cells cultured in androgen-ambient conditions, androgen-deprived cells showed elevated levels of GalNAc (Fig. 5d, Supplementary Fig. S7a–d). This was accompanied by elevated real-time binding of the rVAR2 lectin to LNCaP cells under AD conditions (Supplementary Fig. S8). Moreover, the density (thickness) of GalNAc-containing glycocalyx was increased in LNCaP cells cultured in AD conditions as compared to cells cultured with androgens (Fig. 5e). We also examined the reorganization of the GalNAc-containing glycocalyx along transverse slices from confocal 3D image reconstructions at the interaction point between the cell membrane and the ECM (cell basement). LNCaP cells cultured in AD conditions reorganized the GalNAc-containing glycocalyx into foci-like structures at the cell basement (Fig. 5f, Supplementary Fig. 7d). Combined, these data indicate that CS is a key component of the GalNAc-containing prostate cancer glycocalyx that undergoes morphological reformation after ARPI.

We next investigated the impact of CS upregulation in prostate cancer progression after ARPI using wildtype LNCaP and LNCaPCHase cells. Indeed, LNCaP and LNCaPCHase cells had a similar cell proliferation profile in vitro under ambient androgen conditions (Fig. 5g). However, under AD conditions LNCaPCHase cell proliferation was decreased as compared to wildtype LNCaP cells (Fig. 5g). We next examined the role of CS in prostate cancer cell motility and its ability to promote metastasis. We performed a wound healing assay with wildtype LNCaP and LNCaPCHase cells cultured under ambient androgen or AD conditions. To eliminate differences in proliferation as a confounder for the readout, cells were pretreated for 2 h at time 0 with 2 μg/mL mitomycin C that inhibits cell cycle progression. While wildtype LNCaP cells closed the wound efficiently in both ambient and AD conditions, LNCaPCHase cells failed to close the wound in AD conditions specifically (Fig. 5h and Supplementary Fig. S9a). We next investigated the ability of wildtype LNCaP and LNCaPCHase cells to invade through an artificial ECM mesh ± AD using IncuCyte® ClearView Chemotaxis assay. While wildtype LNCaP and LNCaPCHase cells showed proficient and similar invasion profiles under ambient conditions, LNCaPCHase cells failed to invade specifically under AD (Fig. 5i). As such, CS is required for sustained motility and invasion capacity of prostate cancer cells in vitro after AD. We next investigated the requirement of CS in a xenograft castration model of CRPC progression using wildtype LNCaP and LNCaPCHase cells (Fig. 5j). LNCaPCHase tumors displayed a similar growth pattern to wildtype LNCaP tumors under ambient conditions (Fig. 5k) and were highly positive for CHase (Supplementary Fig. S9b). However, while LNCaP tumors relapsed and progressed into CRPC after castration, LNCaPCHase tumors did not (Fig. 5l). While both castrated LNCaP and LNCaPCHase tumors at experimental endpoint showed an increase in AR mRNA levels (Supplementary Fig. S9c), the difference in AR protein levels was not significant between the different arms (Fig. 5m and Supplementary Fig. S9d), reflecting the complexity and phenotypic diversity of the PC relapsed tumors. However, while there was no significant difference in PSA levels between LNCaP and relapsed castrated LNCaP; castrated LNCaPCHase tumors showed a significant lower level of PSA when compared to uncastrated LNCaPCHase (Fig. 5n and Supplementary Fig. S9d). This is corroborated by RNAseq of additional LNCaP and LNCaPCHase tumors ± castration, showing that while AR mRNA levels increase after castration in both LNCaP and LNCaPCHase tumors, mRNA of established AR target genes *KLK3*/PSA, *TMPRSS2*, *FKBP5*, is significantly decreased only in LNCaPCHase tumors after castration (Fig. Supplementary Fig. S9e). These data indicate that progression to CRPC depends on elevated CS expression.

### The GalNAc-containing glycocalyx supports cell motility and metastases in AR-negative CRPC

**Fig. 6:**
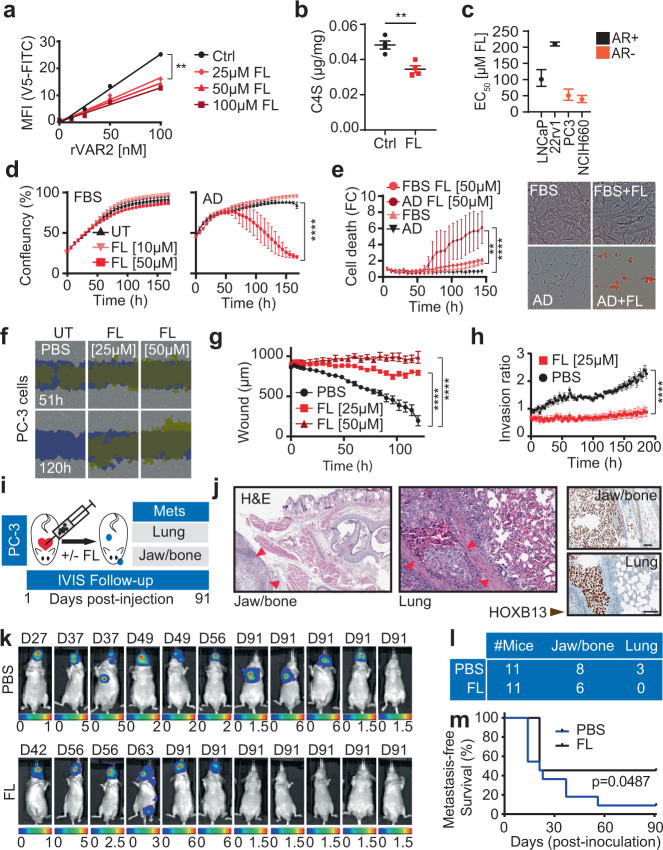
Chondroitin sulfate enables cell motility and metastases in castration-resistant prostate cancer. **a** CSA measurements in LNCaP cells after 72 h of Fluorosamine (FL) treatment using rVAR2 binding on flow cytometry; linear regression followed by Ancova test; *p* < 0.0001, *F* = 18.59, DFn = 3, DFd = 12. **b** LC-MS quantification of C4S in LNCaP cells in AD condition after 72 h of FL treatment (50 μM). Data are shown as mean ± SEM; *n* = 4 biological replicates; *p* = 0.0041 by two-tailed *t* test. **c** FL EC50 values in AR + and AR- PC cell lines. Data are shown as EC50 plus upper and lower limits; *n* = 8 biological replicates. **d** Growth (IncuCyte) of LNCaP cells in FBS or AD conditions ± FL treatments. Data are shown as mean ± SEM, *n* = 4 biologically independent cell cultures; after 100 h *p* < 0.0001 by two-way ANOVA followed by Tukey’s test. **e** Cell death measurement of LNCaP cells treated as in **d** using propidium iodide (red staining; Incucyte) (Left panel). Data are shown as mean ± SEM, *n* = 4 independent samples; after 100 h *p* < 0.05 by two-way ANOVA followed by Tukey’s test. Representative image of Incucyte at endpoint (Right panel) **f** Representative images of wound healing closure in PC3 exposed to FL treatments, yellow show original scratch wound limits. Blue shows closed wound margins. **g** Scratch width measurements of **f** in μm. Data are shown as mean ± SD, *n* = 9 biologically independent replicates; after 6 h *p* < 0.001 by two-way ANOVA followed by Tukey’s test. **h** Invasion capacity (IncuCyte; Chemotaxis assay) of PC3 in presence and absence of FL treatment. Results are expressed as phase object count normalized to initial top value and shown as mean ± SD, *n* = 3 biologically independent replicates; after 26 h *p* < 0.05, after 46 h *p* < 0.0001 by two-way ANOVA followed by Tukey’s test. **i** Protocol schematic of PC3 metastatic seeding capacity in mice. **j** Representative H&E and HOXB13 staining of PC3 bone and lung metastases. Scale bar represents 100 μm **k** IVIS imaging of mice for metastases detection at humane or experimental endpoint. D is the day of termination. **l** Number of mice with jaw/bone or lung metastases in PBS or FL treated group. **m** Kaplan–Meier estimates of metastasis-free survival of mice injected with PC3 cells pretreated with FL 25 μM or Mock; Log-rank test. ns: not significant; **p < 0.01; ****p < 0.0001. Source data are provided as a Source Data file.

We next assessed the importance of the GalNAc-containing glycocalyx for prostate cancer cell motility and metastases in the absence of AR signaling. For this, we used the glucosamine mimic peracetylated 4-F-GlcNAc (Fluorosamine; FL), which is a fluorinated analog of GlcNAc that cannot be converted to GalNAc by the 4-epimerase, resulting in GalNAc depletion^63^. We first assessed if FL would decrease CS expression in prostate cancer cells. As expected, FL treatment reduced CSA chains and C4S GalNAc residues in prostate cancer cells in vitro as detected by live-cell binding of rVAR2 (Fig. 6a) and liquid chromatography (LC)-mass spectrometry (MS) (Fig. 6b). We then established the 50% effective concentration (EC_50_) of FL in four prostate cancer cell lines of which two were AR-driven (LNCaP and 22Rv1) and two AR-independent (PC-3 and NCIH660), using viability as the readout. The AR-independent cells were two to four times more sensitive to FL than the AR-dependent cells (Fig. 6c), supporting the idea that prostate cancer cells become dependent on CS after ARPI. We next analyzed the sensitivity of androgen-dependent LNCaP cells to sub-EC_50_ concentrations of FL ± AD. In ambient hormone conditions, LNCaP cells treated with 10 and 50 µM FL maintained a similar growth pattern as untreated cells (Fig. 6d, left). However, when LNCaP cells were cultured in AD conditions, 50 µM FL induced cell death (Fig. 6d, right and Fig. 6e). Combined, these data maintain that the GalNAc-containing glycocalyx is important for growth and survival of prostate cancer cells after ARPI.

We next wanted to assess the importance of the GalNAc-containing glycocalyx in metastasis formation. To study this, we used a metastatic CRPC cell line PC3-LUC that does not express AR. When treated with FL, PC3-LUC cells behaved similar to LNCaP^CHase^ cells in AD conditions and were unable to migrate (Fig. 6f, g) and invade through an artificial ECM (Fig. 6h). We next investigated the colonization capacity of PC3-LUC cells in vivo+/− pre-treatment with FL. Injection of PC3-LUC cells in the left ventricular compartment of the mouse heart, gives rise to ample metastasis formation in the jaw/bone and lungs (Fig. 6i, j). However, pre-treatment of PC3-LUC cells with FL, delayed the time to humane endpoint, reduced the overall number of mice developing metastasis (Fig. 6k, l), and improved metastasis-free survival (Fig. 6m). Taken together, these data suggest that the GalNAc-containing glycocalyx is functionally important for prostate cancer cell motility and metastasis when AR signaling is lost or inhibited.

## Discussion

The new generation of potent AR pathway inhibitors is currently first-line therapy for men with AR-active metastatic CRPC. However, in about 20% of prostate cancers, these treatments may trigger differentiation of adeno-CRPC towards aggressive anaplastic and neuroendocrine tumors that are associated with loss of canonical AR activity. At this stage, patients have limited therapeutic options and the median overall survival is less than 1 year from the time of diagnosis^64,65^. Therefore, a deeper understanding of how CRPC is able to progress in the absence of AR activity is essential for the development of new therapies. Most studies on AR-independent CRPC have focused on identification of genetic drivers affecting intrinsic cell survival mechanisms such as FGF, BRN2, PEG10, and others^37,46,47,66^. Furthermore, studies on signaling events involving the cellular tumor microenvironment (e.g., endothelial cells, immune cells, and fibroblasts) have added to our understanding of CRPC progression^67–74^. Another but less studied component of the CRPC tumor microenvironment is the ECM and glycocalyx that consist of cellular and extracellular glycoproteins, glycosaminoglycans, and rigid structural macromolecules such as collagen^18–24^. CS is one of the most abundant glycosaminoglycans in prostate tumors and the amount of chondroitin sulfate is increased in cancerous as compared to benign prostate^28^. In tumors, the majority of CS is highly 4-*O*-sulfated, resembling the type of CS found in the human placenta^52,75^. Accordingly, highly 4-*O*-S-sulfated oncofetal CS is currently being developed as a receptor and biomarker for therapeutic and diagnostic applications^26,52,75,76^. Despite decades of observations implicating CS in cancer, the function of CS in tumor progression and therapeutic resistance is not well understood. Here we describe an essential role for CS in growth and survival of aggressive AR-indifferent CRPC and devise a targeting strategy for CRPC based on synthetic GalNAc sugar mimics and enzymatic degradation of CS.

In this work, we report that C4S is upregulated in prostate tumors after the loss of AR signaling and that this event supports survival, proliferation, and metastases formation in AR-indifferent CRPC. Specifically, androgen deprivation led to a profound reformation of the GalNAc glycocalyx that was required for prostate cancer cell survival in vitro and for tumor growth in vivo after ARPI. Forced degradation of CS in androgen-dependent LNCaP tumors by CHase expression, prevented tumor relapse after castration. Moreover, inhibition of GalNAc synthesis by the sugar mimic fluorosamine, was able to reduce CS content and inhibit cell motility and metastasis formation of AR-negative metastatic CRPC cells in vivo.

The enzymes able to catalyze C4S-sulfation of CS chains are CHST11, CHST12, CHST13, and in some contexts CHST14^77^. To identify the enzymes responsible for C4S upregulation in AR-indifferent CRPC, we interrogated RNA-Seq data obtained from the LTN331/LTN331R PDX trans-differentiation model during progression from adenocarcinoma to AR-independent NEPC. Here we identified *CHST11* as >100-fold upregulated on mRNA levels after murine castration. This upregulation of *CHST11* after AR inhibition or loss was confirmed in three independent human prostate tumor cohorts (VPC, IST, and UW cohorts) of 435 patients combined. Mechanistically we found that AR interacted with a conserved ARE in the first intron of the *CHST11* gene with a similar avidity as to the ARE in the PSA gene *KLK3*. Under ambient androgen conditions, AR was able to keep *CHST11* expression in check, but upon androgen deprivation, or after castration of mice in vivo, *CHST11* expression was de-repressed, resulting in an increase of C4S GalNAc residues. As CHST11 and CSA upregulation has been observed in multiple cancers not associated with AR signaling, including breast, lung, colorectal, and ovarian cancer patients^52,78^, there are likely other mechanisms of CHST11 and CSA regulation remaining to be elucidated. It is noteworthy to mention that functional splice variants of CHST11 may exist but these potential variants remain uncharacterized.

In summary, our work establishes CS as an essential component of the GalNAc-containing prostate tumor glycocalyx that supports progression of AR-indifferent CRPC. Our work augments that inhibition of tumor-associated CS would be beneficial to CRPC patients that progress on ARPI and for whom no long-term effective treatments are currently available.

## Methods

### Cell culture

All human cell lines excluding IGR-CaP1 cells were purchased from ATCC. IGR‐CaP1 (RRID: CVCL_A018) cells were provided by ref. 79. All cell lines were confirmed to be free of mycoplasma contamination and were maintained at 37 °C with 5% CO_2_.

LNCaP (ATCC Cat# CRL-1740, RRID:CVCL_1379) and its derivatives, 22Rv1 (ATCC Cat# CRL-2505, RRID:CVCL_1045), and IGR-CaP1 cells were maintained in RPMI 1640 medium containing 10% fetal bovine serum (FBS) (Gibco Cat# 11875119 & Gibco Cat# A3160401). PC3 (ATCC Cat# CRL-1435, RRID:CVCL_0035), VCaP (ATCC Cat# CRL-2876, RRID:CVCL_2235), and DU145 (ATCC Cat# HTB-81, RRID:CVCL_0105) cells were maintained in Dulbecco’s Modified Eagle Medium (DMEM) containing 10% FBS (GE Healthcare Cat# SH30243.01). NCI-H660 (ATCC Cat# CRL-5813, RRID:CVCL_1576) cells were maintained in RPMI 1640 medium containing 5% FBS, 1X Insulin-Transferrin-Selenium (Gibco Cat# 41400045), 10 nM Hydrocortisone, 10 nM beta-estradiol, and 4mM L-glutamine (add 2 mM extra to the media) (Gibco Cat# 35050061). HEK293T (ATCC Cat# CRL-11268) cells were maintained in BalanCD HEK293 medium (Irvine Scientific cat# 91165) supplemented with 8 m ML-glutamine (ATCC 30-2214) and 10 µL/mL of ITS (Corning cat# 25-800-CR). Cell line authentication of LNCaP and its derivative cell line LNCaP-chABC was done by Genetica DNA Laboratories.

### LNCaP^CHase^ cell line generation

A plasmid containing an optimized chondroitinase ABC (CHase) sequence was generously provided by Dr. Elizabeth M. Muir^80^. Pipe cloning was used to insert the CHase sequence into the pLenti-C-Myc-DDK-p2a-puro lentiviral gene expression vector (Origene Cat# PS100092) as previously described^81^.

CHase lentivirus for transduction was produced by transfecting 4 × 10^6^ HEK293T cells with 6 µg pLenti-C-Myc-DDK-p2a-puro-chABC using X-tremeGENE HP DNA transfection reagent (Millipore Sigma Cat# 6366236001) as per manufacturer’s protocols. 48 h post transfection, viral supernatant was collected, centrifuged at 200 g for 10 min and filtered through a 0.45 µm Steriflip (Millipore Sigma Cat# SE1M003M00) to remove cellular debris. 5 × 10^6^ LNCaP cells were plated in six well plate and 18 h later, cells were transduced by replacing culture media with 1.5 mL of viral particle and 1.5 mL serum-free RPMI media, followed by Polybrene Infection/Transfection Reagent (Millipore Sigma Cat# TR-1003-G) to a final concentration of 8 µg/mL. Cells were re-transduced 24 h and 48 h after and CHase cells were selected with 5 µg/mL puromycin (Thermo Fisher Cat# A1113802).

### Western blotting analysis

Western blotting assays were performed as previously described^82^. All antibodies used in the present study, their catalog numbers, applications, and used dilution can be found in Supplementary Table 1. Anti-rabbit and anti-mouse IgG secondary antibodies were purchased from LI-COR. Membranes were scanned using the LI-COR Odyssey® Gel Imaging system. W.B intensities were determined using ImageJ software.

### Quantitative real-time PCR

RNA was extracted from cells and tumor tissues using TRIzol™ Reagent (Invitrogen Cat# 15596018) according to the manufacturer’s protocol. Purified RNA was quantified using ThemoFisher’s NanoDrop Spectrophotometer and 2 µg of RNA was reverse-transcribed into cDNA using M-MLV Reverse Transcriptase (Invitrogen Cat# 28025021) as per manufacturer’s protocol. The resulting cDNA was amplified with SYBR Select Master Mix (Applied Biosystems Cat# 4472920) and gene-specific forward and reverse primers (3 µM) for 50 cycles in the ABI ViiA™ 7 Real-Time PCR system (Thermo Fisher). Gene expression levels were normalized to GAPDH and RPL32 expression. A list of all primers used in the study can be found in Supplementary Table 2.

### Proliferation assay

Cells were plated on a 96-well plate (5000 cells per well) in RPMI 1640 medium containing 10% FBS or in phenol-free RPMI medium containing 10% CSS (Gibco Cat# 11835030 & GE Healthcare Cat# SH30068.03). Cell growth was monitored using the IncuCyte Live-Cell Analysis System (Sartorius). Propidium iodide (500 nM) was added to the media to follow cell death.

Ec50 values were calculated using a weighted five-parameters logistic regression and statistical analyses were performed using GraphPad Prism 8 (GraphPad Software Inc.).

### Ectopic expression of AR

Ectopic expression of AR was achieved by transient transfection of PC-3, with overexpression vectors where wild-type AR under the control of the CMV promoter in the pcDNA3.1 vector (Invitrogen). Empty pcDNA3.1 vector served as a negative control. PC3 cells were seeded in six-well plates (50,000 cells/well) in RPMI 1640 medium with 5% CSS for 24 h, followed by transfection with 0.5 µg or 2 µg using transfection reagent (TT20, Mirus) for 72 h.

### Wound-healing assay

20,000 cells were plated on a poly-L-lysine coated 96-well ImageLock plate (Essen BioScience Cat# 4379). At 80% confluence, cells were treated with 2 µg/ml of Mitomycin C for 2 h. The 96-pin IncuCyte Wound Maker Tool was then used to make a uniform scratch in all the wells of the plate (Essen BioScience Cat# 4563). The media containing Mitomycin C and detached cells was removed and replaced with fresh media containing the appropriate treatment. Cell migration was monitored using the IncuCyte Live-Cell Analysis System (Sartorius).

### Chemotaxis assay

Chemotaxis assays were conducted using the IncuCyte ClearView 96-well cell migration plate (Sartorius Cat# 4582) according to the manufacturer’s instructions (Essen Bioscience Ltd., Hertfordshire, UK). Briefly, 5000 cells plus the treatment in a total volume of 60 μl 1% FBS or CSS medium were added into the ClearView 96-well insert. Each cell plate was then left to settle at the room temperature for 15 min followed by incubation for a further 30 min at 37 °C. 200 μl of medium containing 10% FBS or 10% CSS (chemoattractant) was added to each well of the reservoir plate. The 96-well insert was carefully transferred to the reservoir plate. The ClearView plate was then placed onto the IncuCyte S3® instrument and left for 15 min at 37 °C to settle. After careful removal of any condensation on the lid and bottom of the reservoir, each plate was scanned on the IncuCyte S3 instrument with a 10× objective using the IncuCyte™ chemotaxis system. Chamber wells were analyzed every 3 h using the IncuCyte chemotaxis software.

### Bio-layer interferometry assay

The direct interaction between biotinylated DNA binding domain of the androgen receptor (AR) (residues 556-629) and *CHST11* DNA sequences containing various androgen response elements (ARE) was quantified with biolayer interferometry (BLI) technique using OctetRED (ForteBio). AR-DBD was biotinylated in situ using an AviTag™ sequence (GLNDIFEAQKIEWHE) (Avidity, LLC, Aurora, CO, USA) incorporated at the N-terminus of the protein. Escherichia coli BL21 containing both biotin ligase and AR-DBD vectors were induced with 0.5 mM isopropyl-β-D-1-thiogalactopyranoside and the protein was expressed for 4 h at 20 °C in the presence of 125 µM biotin. Cells were collected and then lysed by sonication, and the resulting lysate was purified by immobilized metal ion affinity chromatography with nickel–nitrilotriacetic acid resin followed by size exclusion chromatography (superdex s75, GE Healthcare). The purified AR-DBD at 0.1 mg/mL was immobilized on the streptavidin sensors (SA) overnight at 4 °C. The sensors were then blocked, washed, and moved into wells containing 10 µM of the studied DNA sequences in reaction buffer (20 mM Tris, pH 8, 150 mM NaCl, 5% glycerol, 2 mM DTT, 0.1 mM PMSF). PSA-ARE (5′-TTA GCT AGC ACT TGC TGT TCT GCA AGT-3′) was used as a positive control and MYC Ebox (5′-TGA AGC AGA CCA CGT GGT CGT CTT CA-3′) was used as a negative control. The sequences of tested *CHST11* sequences are: CHST11-ARE (5′-CTT TTC TAG AAC ACT GGG GCA TCT CCA-3′), CHST11-ARE-mutated (5′-CTT TTC TAT AAT ACT GGT GTA TCT CCA-3′) and CHST11-ARE-deletion (5′-TAT TTG CTT TTC TCT GGG GCA TCT CCA-3′).

### Chromatin immunoprecipitation (ChIP)

LNCaP cells were plated in their respective growth media. Once they reached 40–50% confluency, cells were washed with PBS and placed in RPMI 1640 medium (phenol red-free) containing 10% Charcoal Stripped Serum (CSS) (Gibco Cat# 11835055 & GE Healthcare Cat# SH30068.03) for 72 h and then half the cells were treated with 10 nM R1881 for 24 h. Cells were fixed in 1% PFA and 0.125 M glycine for 10 min and scraped off the plate in cold PBS containing protease inhibitors. Cells pellets were collected and flash frozen in nitrogen. Samples underwent all steps as described in the Magna ChIP A protocol (Millipore Sigma Cat# 17610). Pull-downs were performed using AR antibody (D6F11) (Cell Signaling Cat# 5153S) or IgG antibody (Diagenode Cat# C15410206) followed by qPCR in the ABI ViiA™ 7 Real-Time PCR system (Thermo Fisher). All ChIP primers used in the study can be found in Source Data Table 2.

### Immunofluorescence microscopy

Cells were plated on glass coverslips and treated as reported. Cells were fixed with 4% PFA for 20 min, washed in PBS, and permeabilized in 0.1% saponin with 3% BSA in PBS for 1 h. Fixed cells were incubated with primary antibodies at 4 °C, washed three times with PBS, and incubated in Alexa Fluor 488 conjugated secondary antibodies (Thermo Fisher) for 1 h at room temperature in the dark. After PBS washing cells slides were mounted using DAPI Vecta-shield mounting media (Vector Laboratory Cat# H120010). Images were acquired at 60× magnification on the Olympus FV3000RS or Zeiss LSM 780 confocal microscopes. Carl Zeiss (zen) software was used for confocal microscopy analysis.

### Glycocalyx imaging

Cells were seeded on glass coverslips and treated with 40 µM of GlcNAz (Thermo Fisher Cat# PI88905) for 48 h. After three washes with cold DPBS (Gibco Cat# A1285801), cells were Cu-click conjugated with AlexaFluor-488-alkyne (Click Chemistry Tools Cat# 12771) and fixed as previously reported (^83^). Cells were mounted onto glass slides with ProLong glass mounting reagent (Thermo Fisher Cat# P36980). Images were acquired at 60× magnification on the Olympus FV3000RS confocal microscope.

The glycocalyx quantification was determined by defining a region of interest around individual cells and then measuring the intensity of the staining in each region using ImageJ software. Intensity plot of the cell membrane was generated and the width at half maximum intensity was considered as glycocalyx thickness.

### In vivo mouse models

Male *Nu/Nu* mice were purchased from ENVIGO and housed in the animal care facility at the Vancouver Prostate Centre. All animal studies were performed in accordance with protocols approved by the Animal Care Committee at the University of British Columbia (A19–0324). Mice were maintained in ventilated cages (4 mice per cage), with constant humidity (25–47%) and temperature (21–22 °C), under a 12 h:12 h light:dark cycle, and had ad libitum access to rodent chow diet and drinking water.

LNCaP or LNCaP^chABC^ cells (2 × 10^6^ cells) suspended in PBS and Matrigel® Matrix solution (1:1) (Corning Cat# 356234) were injected subcutaneously into the right flank of 6-week-old Nu/Nu male. Once tumors reached 100–150 mm^3^ in volume, mice were paired together based on similar tumor size and one mouse in each pair was castrated. Tumor growth was monitored weekly using a caliper-measuring tool. The tumor volume was calculated using ellipsoid volume formula; *V* = (4/3)π(*L*/2)*(*W*/2)*(*D*/2) and mice were euthanized before reaching the maximal tumor size of 1000 mm^3^.

PC3-LUC cells were pre-treated with 25 µM of Fluorosamine (Synthesized by Life Chemicals as previously described^84^) for 72 h and collected using CellStripper dissociation reagent. 1 × 10^6^ PC3-LUC cells suspended in 100 µl of PBS were injected into the left ventricle of 6-week-old Nu/Nu male mice under ultrasound guidance using a 30-gauge needle. Metastases formation was monitored using IVIS spectrum CT scanner (Perkin Elmer) and analyzed using IVIS Living Image Software. Metastasis sites were collected and fixed in formalin for pathology studies at humane or experimental endpoint.

### Extraction and isolation of GAGs for mass spectrometry

Paraffin-embedded (FFPE) tissues were obtained from Vancouver Prostate Centre Tissue Bank. The study protocol was approved by University of British Columbia Clinical Research Ethics Board and Vancouver Coastal Health Research Institute Research Ethics Board.

Tissue cores were extracted from paraffin blocks, deparaffinized using xylene, and rehydrated overnight in PBS at 4 °C. Samples were weighed and lysed with beads in lysis buffer and protein concentration was determined using BCA assay.

The homogenized tissue was treated with 0.4 mg/mL Pronase (Sigma from Streptomyces griseus) and 0.1% Triton X-100 at 37 °C, shaking, overnight. For GAG isolation, a 0.5 mL bed volume DEAE Sephacel (GE-Healthcare) column was prepared. The column was equilibrated in equilibration buffer (50 mM NaOAc, 0.2 M NaCl, 0.1% Triton X-100, pH 6.0) and the sample supernatant was added, after centrifuging them at 20,000 *g* for 10 min. The columns were washed in 20 mL wash buffer (50 mM NaOAc, 0.2 M NaCl, pH 6.0) and samples were eluded in 2 mL 2 M NaCl. After elution, the samples were precipitated in ethanol saturated with NaOAc (1:3, V:V) at 20,000 *g*, 4 °C, for 20 min. Leftover ethanol was evaporated in a centrifugal evaporator and the remaining GAGs were reconstituted in 100 µL DNase buffer (50 mM Tris, 50 mM NaCl, 2.5 mM MgCl2, 0.5 mM CaCl2, pH 8.0). The samples were digested with 20 kU/mL DNaseI (Sigma D-4263 from Bovine Pancreas) for 2 h at 37 °C, shaking, and then β-eliminated at a concentration of 0.4 M NaOH overnight, 4 °C. The samples were neutralized with acetic acid, and the GAGs were purified on a DEAE column and ethanol precipitated as described previously.

Glycans extraction from cell pellets: After 2× PBS washes, cells were detached using CellStripper Dissociation Reagent (Fisher Scientific Cat# MT20256CI), counted and pellets of 3 million cells were collected. Pellets were treated with 0.25U of chABC enzyme (Sigma Cat# C2905) in 50 µl of chABC buffer for 2 h at 37 °C on a rotor. After chABC treatment, protein concentration of cell pellets was determined for normalization when appropriate. When comparing different cell lines, cell numbers were used for normalization.

### LC-MS analysis

Samples were digested with 20 mU Chondroitinase ABC (Sigma C3667) for 2 h at 37 °C and dried in a centrifugal evaporator. The digested polysaccharides were subjected to reductive amination using 17 µL [^12^C_6_] aniline (Sigma Aldrich 242284) and 17 uL reducing reagent containing 1 M NaCNBH3 (Sigma) in dimethyl sulfoxide acetic acid (7:3, V/V). The [^12^C_6_] aniline tagged samples were mixed with 20 ρmole [^13^C_6_] aniline-labeled standards and analyzed by GRIL-LC MS/MS. The samples were separated on a C-18 reverse phase column (TARGA C18, 150 mm × 1.0 mm, 5 µm, Higgins Analytical, Inc.) with 5 mM of the ion pairing agent dibutyl amine (Sigma). The ions were monitored in negative mode using the same gradient, capillary temperature, and spray voltage as described previously^85^. The analysis was done on an LTQ Orbitrap Discovery electrospray ionization mass spectrometer (Thermo Scientific) equipped with an Ultimate 3000 quaternary HPLC pump (Dionex).

### Flow cytometry

Cells were grown to 70–80% confluency in appropriate growth media and harvested in CellStripper. Cells were incubated with rVAR2 protein (200–25 nM) in PBS containing 2% FBS for 30 min at 4 °C and binding was acquired in a FACS Calibur (BD Biosciences) after a secondary incubation with an anti-V5-FITC antibody. Single cells were gated using FSC-H/FSC-A parameters. FlowJo (v9) and Cyflogic (1.2.1) softwares were used for FACS analysis Gating strategy example in Source Data file

### Immunohistochemistry (IHC)

HOXB13 and chABC expression was assessed in xenograft tissue using Ventana DISCOVERY Ultra autostainer (Ventana Medical Systems, Tucson, Arizona). After baking and deparaffinization, tissue sections were incubated in Tris-based buffer (CC1, Ventana) at 95 °C for 64 min to retrieve antigen, followed by primary antibody incubation at room temperature: HOXB13 (1:500, #90944, Cell Signaling Technologies, rabbit, monoclonal (D7N80), incubation: 1 h); chABC (1:100, NBP1-96141H, Novus Biological mouse, monoclonal (1E10), incubation: 12 h). Primary antibodies bound to HOXB13 were visualized with UltraMap DAB anti-Rb Detection Kit (Ventana), while primary antibodies against ChABC were incubated with unconjugated rabbit anti-rat IgG (H + L) (312-005-045, Jackson ImmunoResearch Laboratories) and detected with DISCOVERY HQ-HRP detection (Ventana). Stained xenograft sections were digitized with Leica SCN400 scanner (Leica Microsystems; Concord, Ontario, Canada) at magnification equivalent to 20×.

### Electrophoretic mobility shift assay

20 µM F and R oligos were annealed at 95 °C for 10 min in a heat block then allowed to cool slowly in the heat block overnight to room temperature. CHST11-ARE primers: F: /AG ATG CCC CAG TGT TCT AG; R: /CT AGA ACA CTG GGG CAT CT. TMPRSS2-ARE primers: F: /CTAGTTATGAGTACCTGCCGTACCCTTTC; R: /TCGAGAAAGGGTACGGCAGGTACTCATAA. PSA-ARE primers: F: /5IRD700/TACAAATAGGTTCTTGGAGTACTTTACTAGGCATGGACAATG; R: /CATTGTCCATGCCTAGTAAAGTACTCCAAGAACCTATTTGTA.

100 fmol of infrared labeled PSA-ARE and CHST11-ARE annealed oligos were incubated with an increasing amount of AR-DBD recombinant protein in 10 mM Tris /50 mM KCl/1 mM DTT (pH 7.5), 1 µg Poly(dI•dC) in 0.5 mM Tris/50 µM EDTA (pH 7.5), and 2.5 mM DTT/0.25% Tween 20 for 20 min at room temperature in the dark. 10× orange loading dye (LICOR) was added to 1× and EMSA reactions were run in the dark on a 6% native acrylamide gel for 30 min at 100 V in 0.5× TBE. The gel was removed from gel plates and immediately scanned using a LICOR Odyssey Infrared Imaging System. For competitive EMSA, 500 ng AR-DBD recombinant protein were incubated with 20 nmol unlabeled competitor DNA oligos for 5 min at RT in 10 mM Tris /50 mM KCl/1 mM DTT (pH 7.5), 1 µg Poly(dI•dC) in 0.5 mM Tris/50 µM EDTA (pH 7.5), and 2.5 mM DTT/0.25% Tween 20. 100 fmol of IR700-labeled TMPRSS2-ARE DNA oligos were added and incubated for an additional 20 min at RT in the dark.

### Reporting summary

Further information on research design is available in the Nature Research Reporting Summary linked to this article.

## Supplementary information

Supplementary Information

Reporting Summary

## Data Availability

Previously published RNA-seq data that were re-analyzed here are available under accession code https://www.ncbi.nlm.nih.gov/geo/query/acc.cgi?acc=GSE147250; https://www.ncbi.nlm.nih.gov/geo/query/acc.cgi?acc=GSE59986. The Chip-SEQ data reanalyzed here were obtained from Cistrome.com; WashU Epigenome Browser using data from refs. 50; Chng et al. (2012). Source Data are provided in this paper. All other data supporting the findings of this study are available in the Source Data file. All unique biological materials are available by request from the corresponding author. Source data are provided with this paper.

## Source data

Source Data

## References

[1] Davey RA, Grossmann M (2016). Androgen receptor structure, function and biology: from bench to bedside.. Clin. Biochem. Rev..

[2] Karantanos T, Corn PG, Thompson TC (2013). Prostate cancer progression after androgen deprivation therapy: mechanisms of castrate resistance and novel therapeutic approaches. Oncogene.

[3] Cai C (2011). Androgen receptor gene expression in prostate cancer is directly suppressed by the androgen receptor through recruitment of lysine-specific demethylase 1. Cancer Cell.

[4] Augello MA, Den RB, Knudsen KE (2014). AR function in promoting metastatic prostate cancer. Cancer Metastasis Rev..

[5] Perlmutter MA, Lepor H (2007). Androgen deprivation therapy in the treatment of advanced prostate cancer. Rev. Urol..

[6] Kahn B, Collazo J, Kyprianou N (2014). Androgen receptor as a driver of therapeutic resistance in advanced prostate cancer. Int. J. Biol. Sci..

[7] Rice MA, Malhotra SV, Stoyanova T (2019). Second-generation antiandrogens: from discovery to standard of care in castration resistant prostate cancer. Front. Oncol..

[8] Beer TM (2014). Enzalutamide in metastatic prostate cancer before chemotherapy. N. Engl. J. Med..

[9] de Bono JS (2011). Abiraterone and increased survival in metastatic prostate cancer. N. Engl. J. Med..

[10] Ryan CJ (2013). Abiraterone in metastatic prostate cancer without previous chemotherapy. N. Engl. J. Med..

[11] Fizazi K (2019). Abiraterone acetate plus prednisone in patients with newly diagnosed high-risk metastatic castration-sensitive prostate cancer (LATITUDE): final overall survival analysis of a randomised, double-blind, phase 3 trial. Lancet Oncol..

[12] Chi KN (2018). Patient-reported outcomes following abiraterone acetate plus prednisone added to androgen deprivation therapy in patients with newly diagnosed metastatic castration-naive prostate cancer (LATITUDE): an international, randomised phase 3 trial. Lancet Oncol..

[13] Parker CC (2018). Radiotherapy to the primary tumour for newly diagnosed, metastatic prostate cancer (STAMPEDE): a randomised controlled phase 3 trial. Lancet.

[14] Davis ID (2019). Enzalutamide with standard first-line therapy in metastatic prostate cancer. N. Engl. J. Med..

[15] Ku SY, Gleave ME, Beltran H (2019). Towards precision oncology in advanced prostate cancer. Nat. Rev. Urol..

[16] Watson PA, Arora VK, Sawyers CL (2015). Emerging mechanisms of resistance to androgen receptor inhibitors in prostate cancer. Nat. Rev. Cancer.

[17] Beltran H (2014). Aggressive variants of castration-resistant prostate cancer. Clin. Cancer Res..

[18] Chaudhuri O, Cooper-White J, Janmey PA, Mooney DJ, Shenoy VB (2020). Effects of extracellular matrix viscoelasticity on cellular behaviour. Nature.

[19] Liotta LA, Kohn EC (2001). The microenvironment of the tumour-host interface. Nature.

[20] Barnes JM (2018). A tension-mediated glycocalyx-integrin feedback loop promotes mesenchymal-like glioblastoma. Nat. Cell Biol..

[21] Cherfils-Vicini, J. et al. Cancer cells induce immune escape via glycocalyx changes controlled by the telomeric protein TRF2. *EMBO J.* **38**, 10.15252/embj.2018100012 (2019). 10.15252/embj.2018100012 PMC6545744 31000523

[22] Paszek MJ (2014). The cancer glycocalyx mechanically primes integrin-mediated growth and survival. Nature.

[23] Shurer CR (2019). Physical principles of membrane shape regulation by the glycocalyx. Cell.

[24] Woods, E. C. et al. A bulky glycocalyx fosters metastasis formation by promoting G1 cell cycle progression. *Elife* **6**, 10.7554/eLife.25752 (2017). 10.7554/eLife.25752 PMC5739539 29266001

[25] Afar DE (2004). Preclinical validation of anti-TMEFF2-auristatin E-conjugated antibodies in the treatment of prostate cancer. Mol. Cancer Ther..

[26] Agerbaek MO (2018). The VAR2CSA malaria protein efficiently retrieves circulating tumor cells in an EpCAM-independent manner. Nat. Commun..

[27] Clausen TM (2016). Oncofetal chondroitin sulfate glycosaminoglycans are key players in integrin signaling and tumor cell motility. Mol. Cancer Res.

[28] De Klerk DP, Lee DV, Human HJ (1984). Glycosaminoglycans of human prostatic cancer. J. Urol..

[29] Edwards IJ (2012). Proteoglycans in prostate cancer. Nat. Rev. Urol..

[30] Feferman L (2013). Arylsulfatase B (N-acetylgalactosamine-4-sulfatase): potential role as a biomarker in prostate cancer. Prostate Cancer Prostatic Dis..

[31] Feferman L (2017). Arylsulfatase B is reduced in prostate cancer recurrences. Cancer Biomark..

[32] Munkley J (2017). Glycosylation is a global target for androgen control in prostate cancer cells. Endocr. Relat. Cancer.

[33] Munkley, J. et al. Androgen-regulated transcription of ESRP2 drives alternative splicing patterns in prostate cancer. *Elife* **8**, 10.7554/eLife.47678 (2019). 10.7554/eLife.47678 PMC6788855 31478829

[34] Ricciardelli C (1998). Elevated levels of versican but not decorin predict disease progression in early-stage prostate cancer. Clin. Cancer Res..

[35] Sakko AJ (2003). Modulation of prostate cancer cell attachment to matrix by versican. Cancer Res..

[36] Scott, E. & Munkley, J. Glycans as biomarkers in prostate cancer. *Int. J. Mol. Sci.* **20**, 10.3390/ijms20061389 (2019). 10.3390/ijms20061389 PMC6470778 30893936

[37] Akamatsu S (2015). The placental gene PEG10 promotes progression of neuroendocrine prostate cancer. Cell Rep..

[38] Lin D (2015). Identification of DEK as a potential therapeutic target for neuroendocrine prostate cancer. Oncotarget.

[39] Wyatt AW (2014). Heterogeneity in the inter-tumor transcriptome of high risk prostate cancer. Genome Biol..

[40] Thomas MA, Preece DM, Bentel JM (2010). Androgen regulation of the prostatic tumour suppressor NKX3.1 is mediated by its 3’ untranslated region. Biochem. J..

[41] Clinckemalie L (2013). Androgen regulation of the TMPRSS2 gene and the effect of a SNP in an androgen response element. Mol. Endocrinol..

[42] Lucas JM (2008). The androgen-regulated type II serine protease TMPRSS2 is differentially expressed and mislocalized in prostate adenocarcinoma. J. Pathol..

[43] Afar DE (2001). Catalytic cleavage of the androgen-regulated TMPRSS2 protease results in its secretion by prostate and prostate cancer epithelia. Cancer Res..

[44] Magee JA, Chang LW, Stormo GD, Milbrandt J (2006). Direct, androgen receptor-mediated regulation of the FKBP5 gene via a distal enhancer element. Endocrinology.

[45] Gery S, Sawyers CL, Agus DB, Said JW, Koeffler HP (2002). TMEFF2 is an androgen-regulated gene exhibiting antiproliferative effects in prostate cancer cells. Oncogene.

[46] Bluemn EG (2017). Androgen receptor pathway-independent prostate cancer is sustained through FGF signaling. Cancer Cell.

[47] Vlachostergios PJ, Puca L, Beltran H (2017). Emerging variants of castration-resistant prostate cancer. Curr. Oncol. Rep..

[48] Mu P (2017). SOX2 promotes lineage plasticity and antiandrogen resistance in TP53- and RB1-deficient prostate cancer. Science.

[49] Handle F (2019). Drivers of AR indifferent anti-androgen resistance in prostate cancer cells. Sci. Rep..

[50] Chen Z (2015). Agonist and antagonist switch DNA motifs recognized by human androgen receptor in prostate cancer. EMBO J..

[51] Chng, K. R. et al. A transcriptional repressor co-regulatory network governing androgen response in prostate cancers. *EMBO J.* **31**, 2810–2823 (2012). 10.1038/emboj.2012.112 PMC3380210 22531786

[52] Salanti A (2015). Targeting human cancer by a glycosaminoglycan binding malaria protein. Cancer Cell.

[53] Evers MR, Xia G, Kang HG, Schachner M, Baenziger JU (2001). Molecular cloning and characterization of a dermatan-specific N-acetylgalactosamine 4-O-sulfotransferase. J. Biol. Chem..

[54] Mikami T, Mizumoto S, Kago N, Kitagawa H, Sugahara K (2003). Specificities of three distinct human chondroitin/dermatan N-acetylgalactosamine 4-O-sulfotransferases demonstrated using partially desulfated dermatan sulfate as an acceptor: implication of differential roles in dermatan sulfate biosynthesis. J. Biol. Chem..

[55] Smith R (2020). Enzalutamide response in a panel of prostate cancer cell lines reveals a role for glucocorticoid receptor in enzalutamide resistant disease. Sci. Rep..

[56] Mahajan R (2018). Chondroitinase ABC enzyme: a potential treatment option for spinal cord injury.. Int. J. Appl. Basic Med. Res..

[57] Warren PM (2020). Secretion of a mammalian chondroitinase ABC aids glial integration at PNS/CNS boundaries. Sci. Rep..

[58] Lindahl, U., Couchman, J., Kimata, K. & Esko, J. D. Essentials of Glycobiology (eds Ajit Varki, Gerald W. Hart, Jeffrey D. Esko, Richard D. Cummings) 207–221 (2015).

[59] Avram S, Shaposhnikov S, Buiu C, Mernea M (2014). Chondroitin sulfate proteoglycans: structure-function relationship with implication in neural development and brain disorders. Biomed. Res. Int..

[60] Pudelko A, Wisowski G, Olczyk K, Kozma EM (2019). The dual role of the glycosaminoglycan chondroitin-6-sulfate in the development, progression and metastasis of cancer. FEBS J..

[61] Tarbell JM, Cancel LM (2016). The glycocalyx and its significance in human medicine. J. Intern. Med..

[62] Hang HC, Yu C, Kato DL, Bertozzi CR (2003). A metabolic labeling approach toward proteomic analysis of mucin-type O-linked glycosylation. Proc. Natl Acad. Sci. USA.

[63] Nigro J (2009). Regulation of heparan sulfate and chondroitin sulfate glycosaminoglycan biosynthesis by 4-fluoro-glucosamine in murine airway smooth muscle cells. J. Biol. Chem..

[64] Aparicio AM (2013). Platinum-based chemotherapy for variant castrate-resistant prostate cancer. Clin. Cancer Res..

[65] Papandreou CN (2002). Results of a phase II study with doxorubicin, etoposide, and cisplatin in patients with fully characterized small-cell carcinoma of the prostate. J. Clin. Oncol..

[66] Bishop JL (2017). The master neural transcription factor BRN2 is an androgen receptor-suppressed driver of neuroendocrine differentiation in prostate cancer. Cancer Discov..

[67] Calcinotto A (2018). IL-23 secreted by myeloid cells drives castration-resistant prostate cancer. Nature.

[68] Karlou M, Tzelepi V, Efstathiou E (2010). Therapeutic targeting of the prostate cancer microenvironment. Nat. Rev. Urol..

[69] Orr B (2012). Identification of stromally expressed molecules in the prostate by tag-profiling of cancer-associated fibroblasts, normal fibroblasts and fetal prostate. Oncogene.

[70] Tyekucheva S (2017). Stromal and epithelial transcriptional map of initiation progression and metastatic potential of human prostate cancer. Nat. Commun..

[71] Valencia T (2014). Metabolic reprogramming of stromal fibroblasts through p62-mTORC1 signaling promotes inflammation and tumorigenesis. Cancer Cell.

[72] Wan X (2014). Prostate cancer cell-stromal cell crosstalk via FGFR1 mediates antitumor activity of dovitinib in bone metastases. Sci. Transl. Med..

[73] Wu JB (2017). MAOA-dependent activation of Shh-IL6-RANKL signaling network promotes prostate cancer metastasis by engaging tumor-stromal cell interactions. Cancer Cell.

[74] Zhang Z (2020). Tumor microenvironment-derived NRG1 promotes antiandrogen resistance in prostate cancer. Cancer Cell.

[75] Seiler R (2017). An oncofetal glycosaminoglycan modification provides therapeutic access to cisplatin-resistant bladder cancer. Eur. Urol..

[76] Clausen TM (2020). A simple method for detecting oncofetal chondroitin sulfate glycosaminoglycans in bladder cancer urine. Cell Death Discov..

[77] Mikami T, Kitagawa H (2013). Biosynthesis and function of chondroitin sulfate. Biochim. Biophys. Acta.

[78] Oliveira-Ferrer L, Hessling A, Trillsch F, Mahner S, Milde-Langosch K (2015). Prognostic impact of chondroitin-4-sulfotransferase CHST11 in ovarian cancer. Tumour Biol..

[79] Chauchereau A (2011). Stemness markers characterize IGR-CaP1, a new cell line derived from primary epithelial prostate cancer. Exp. Cell Res..

[80] Zhao RR (2011). Lentiviral vectors express chondroitinase ABC in cortical projections and promote sprouting of injured corticospinal axons. J. Neurosci. Methods.

[81] Klock HE, Lesley SA (2009). The Polymerase Incomplete Primer Extension (PIPE) method applied to high-throughput cloning and site-directed mutagenesis. Methods Mol. Biol..

[82] Al Nakouzi N (2016). Clusterin knockdown sensitizes prostate cancer cells to taxane by modulating mitosis. EMBO Mol. Med..

[83] Hong, V., Steinmetz, N. F., Manchester, M. & Finn, M. G. Labeling live cells by copper-catalyzed alkyne–azide click chemistry. *Bioconjug Chem*. **21**, 1912–1916. 10.1021/bc100272z (2010). 10.1021/bc100272z PMC3014321 20886827

[84] Keough MB (2016). An inhibitor of chondroitin sulfate proteoglycan synthesis promotes central nervous system remyelination. Nat. Commun..

[85] Lawrence R (2008). Evolutionary differences in glycosaminoglycan fine structure detected by quantitative glycan reductive isotope labeling. J. Biol. Chem..

