# Supplementary material for: Reformation of the chondroitin sulfate glycocalyx enables progression of AR-independent prostate cancer
Source: Nat Commun. 2022 Aug 13;13:4760. doi: 10.1038/s41467-022-32530-7 (PMC9376089; doi:10.1038/s41467-022-32530-7)
Supplement: Supplementary file 1 — Supplementary Information [file 41467_2022_32530_MOESM1_ESM.pdf]

Supplementary information for

Reformation of the chondroitin sulfate glycocalyx enables progression of AR-independent prostate cancer

Nader Al-Nakouzi, Chris Kedong Wang, Htoo Zarni Oo, Irina Nelepcu, Nada Lallous, Charlotte B. Spliid, Nastaran Khazamipour, Joey Lo, Sarah Truong, Colin Collins, Desmond Hui, Shaghayegh Esfandnia, Hans Adomat, Thomas Mandel Clausen, Tobias Gustavsson, Swati Choudhary, Robert Dagil, Eva Corey, Yuzhuo Wang, Anne Chauchereau, Ladan Fazli, Jeffrey D. Esko, Ali Salanti, Peter S Nelson, Martin E Gleave & Mads Daugaard

Corresponding Authors:
Correspondance should be adressed to Mads Daugaard (mads.daugaard@ubc.ca)

Supplementary Figure 1

a

	AR	KLK3	TMEFF2	NKX3-1	TMPRSS2	FKBP5
HS3ST5	-0.03	-0.27	-0.13	-0.19	-0.16	-0.20
CHST11	-0.14	-0.52	-0.37	-0.46	-0.53	-0.25
HS6ST2	-0.12	-0.07	-0.12	-0.14	-0.16	-0.07
HS3ST3A1	-0.23	-0.20	-0.34	-0.31	-0.30	-0.08
UST	-0.19	-0.20	-0.32	-0.23	-0.13	0.05
B3GAT2	-0.09	-0.24	-0.04	-0.23	-0.20	-0.24
HS3ST1	0.10	-0.29	-0.14	-0.03	-0.16	0.01
HS6ST3	0.26	-0.09	-0.02	0.04	-0.11	-0.15
SULT4A1	-0.08	-0.18	0.18	-0.11	-0.22	-0.18
CHST3	-0.11	-0.33	-0.34	-0.42	-0.35	-0.08
HS3ST3B1	-0.18	-0.27	-0.28	-0.39	-0.29	-0.22
LIPE	-0.12	-0.37	-0.06	-0.39	-0.32	-0.28
DSEL	0.20	-0.12	0.39	0.04	0.03	-0.15
GLCE	-0.05	-0.30	-0.37	-0.10	-0.21	-0.08
EXTL3	-0.04	-0.38	-0.33	-0.35	-0.37	-0.18
EXT1	0.11	-0.32	-0.23	-0.33	-0.26	-0.27
EXTL2	-0.12	0.46	0.33	0.31	0.45	0.36
XYLT2	0.27	-0.08	0.02	-0.16	-0.14	-0.17
CES3	0.10	-0.22	0.16	-0.05	-0.07	-0.02
CSGALNACT1	-0.12	0.24	0.04	0.21	0.16	0.32
PNPLA7	-0.02	-0.02	0.31	-0.01	0.05	-0.05
PRDX6	0.08	0.40	0.36	0.49	0.31	0.12
GAL3ST2	0.63	-0.16	0.11	0.03	0.04	-0.04
B3GAT1	-0.09	0.07	0.28	0.09	0.05	0.02
SULT1C4	0.03	-0.26	-0.15	-0.34	-0.37	-0.22
CHST15	-0.17	-0.33	-0.38	-0.39	-0.25	0.00
HS3ST4	0.28	0.13	0.50	0.12	0.18	0.24
SULT2B1	-0.19	0.52	0.06	0.46	0.38	0.29
SULT1C2	0.23	-0.14	0.07	-0.11	-0.12	-0.11

b

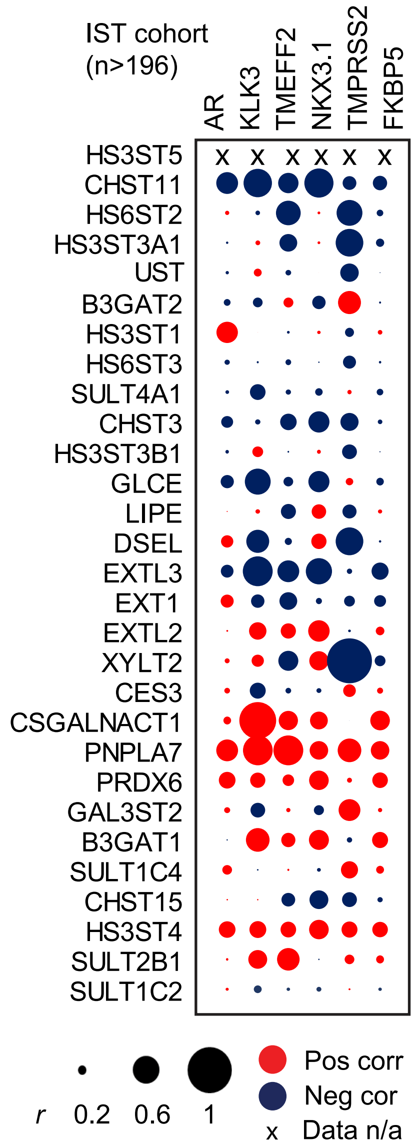

Supplementary Figure 1: Correlation of GAGs genes and AR regulated genes
(a) Pearson correlation coefficient values used in Fig. 2a. (b) Correlation analysis of mRNA levels of GAGs genes identified in Fig.1c and AR regulated genes (*KLK3, TMEFF2, NKX3.1, TMPRSS2, FKBP5*). Circle size represents Pearson correlation coefficient absolute values. Blue, negative correlation; Red, positive correlation, n=196 patients' samples obtained from IST Online (MediSapiens; https://ist.medisapiens.com/).

Supplementary Figure 2

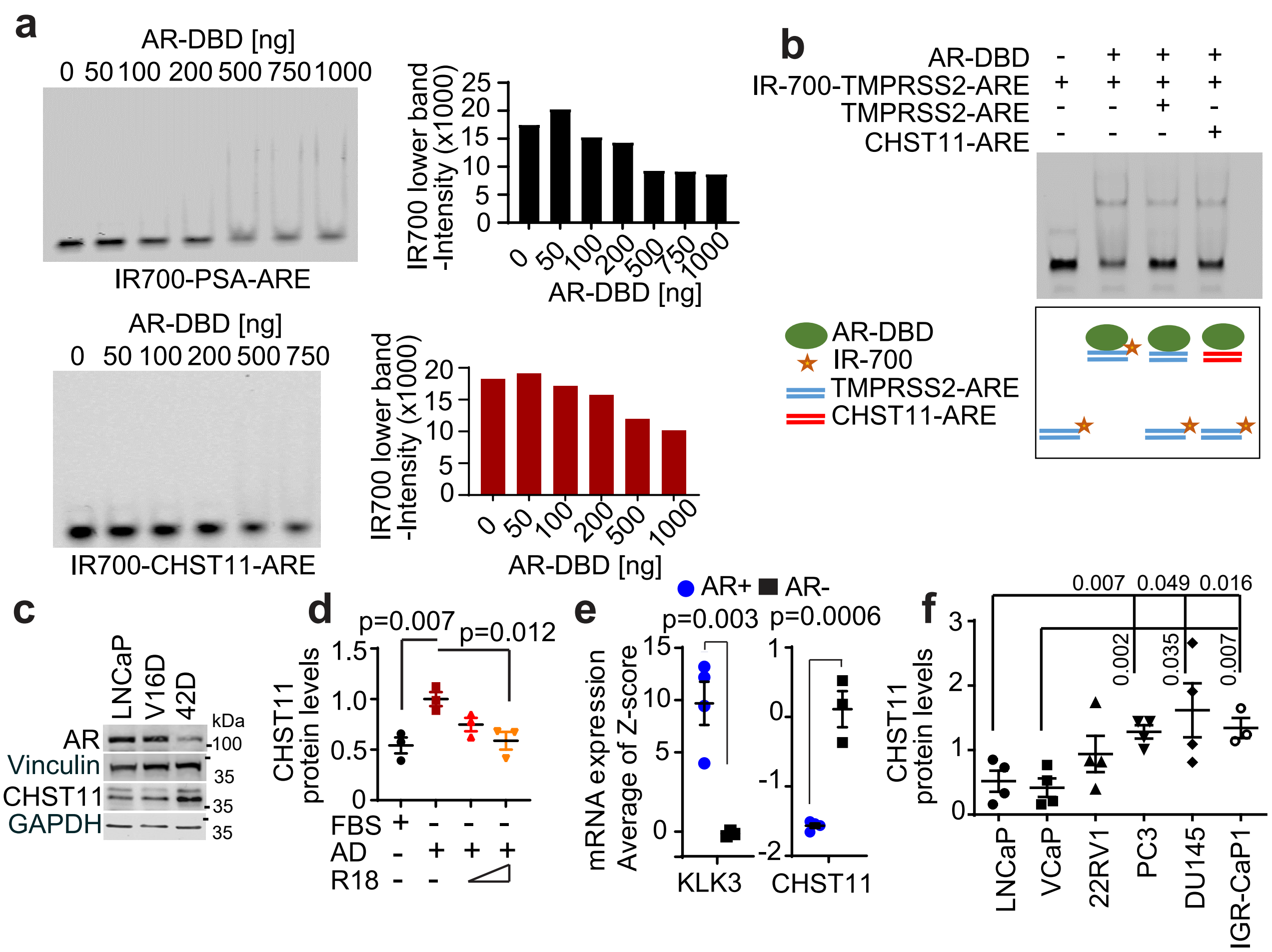

Supplementary Figure 2: AR represses CHST11 expression

(a) EMSA between an increasing amount of AR-DBD recombinant protein and 100 fmol of IR700-CHST11-ARE DNA sequence. IR700-PSA-ARE DNA sequence was used as a reference and a positive control. Amounts of unbound IR700-DNA probes was determined using Image J software. (b) Competitive EMSA between TMPRSS2 and CHST11 probes. Infrared labeled TMPRSS2-ARE probe was incubated with AR-DBD protein in presence and absence of competitor unlabeled CHST11-ARE and TMPRSS2-ARE probes. (c) AR and CHST11 protein expression in LNCaP cells and its derivatives V16D (CRPC) and 42D (NEPC-like). (d) W.B quantification for Fig. 3f. CHST11 immunoblot intensities of three independant experiments were measured using Image J and normalized to loading controls (Actin or vinculin). Data represent intensity mean ± SEM. Significance was tested using ANOVA followed by Tukey's correction. (e) Microarray analysis of mRNA expression (average z-scores from cBioPortal, cancer cell line encyclopedia; Novartis/Broad, Nature 2012) of CHST11 in AR (+) cell lines (n=4 cell lines: LNCaP, VCaP, 22RV1, MDAPCA2b) and AR (-) cell lines (n=3 cell lines: PC3, DU145, NCIH660). Data are presented as mean ± SEM; t-test. (f) CHST11 protein levels in different prostate cancer cell lines. CHST11 immunoblot intensities of four independent experiments were measured using Image J and normalized to GAPDH. Data are presented as mean ± SEM; multiple two tailed t-test. Source data are provided as a Source Data file.

Supplementary Figure 3

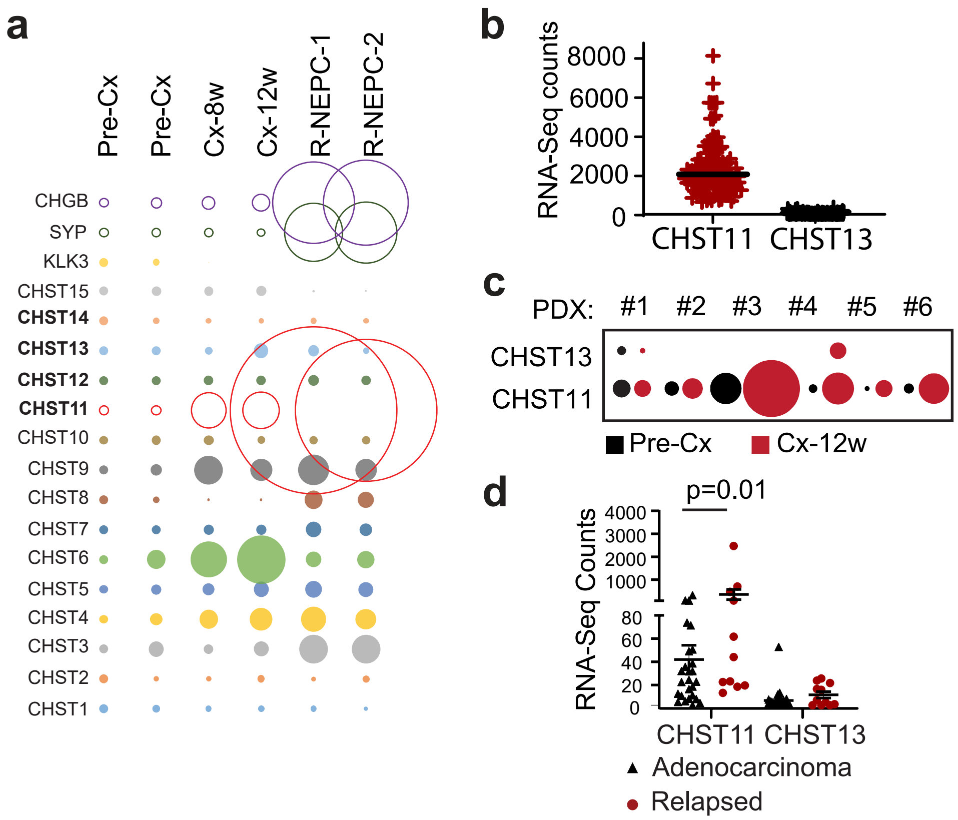

Supplementary Figure 3: CHST expression in PC after castration

(a) CHST sulfotransferases (CHST1 to CHST15), KLK3 and neuroendocrine markers (SYP and CHGB) mRNA expression during transdifferentiation. Circle size represents fold change of RNA-Seq normalized counts compared to average of PreCX condition samples. (b) *CHST11* and *CHST13* gene expression in PC patients (VPC cohort, n=101). (c) *CHST11* and *CHST13* gene expression in six different PC PDXs before (Pre-CX) and 12 weeks after castration (Cx-12w). Circle size represents normalized RNA-Seq counts. (d) RNA-Seq gene expression of CHST11 and CHST13 in Adeno (n=26) and relapsed (n=11) PC PDXs. Data represents mean of normalized RNA-Seq counts ± SEM. Source data are provided as a Source Data file.

Supplementary Figure 4

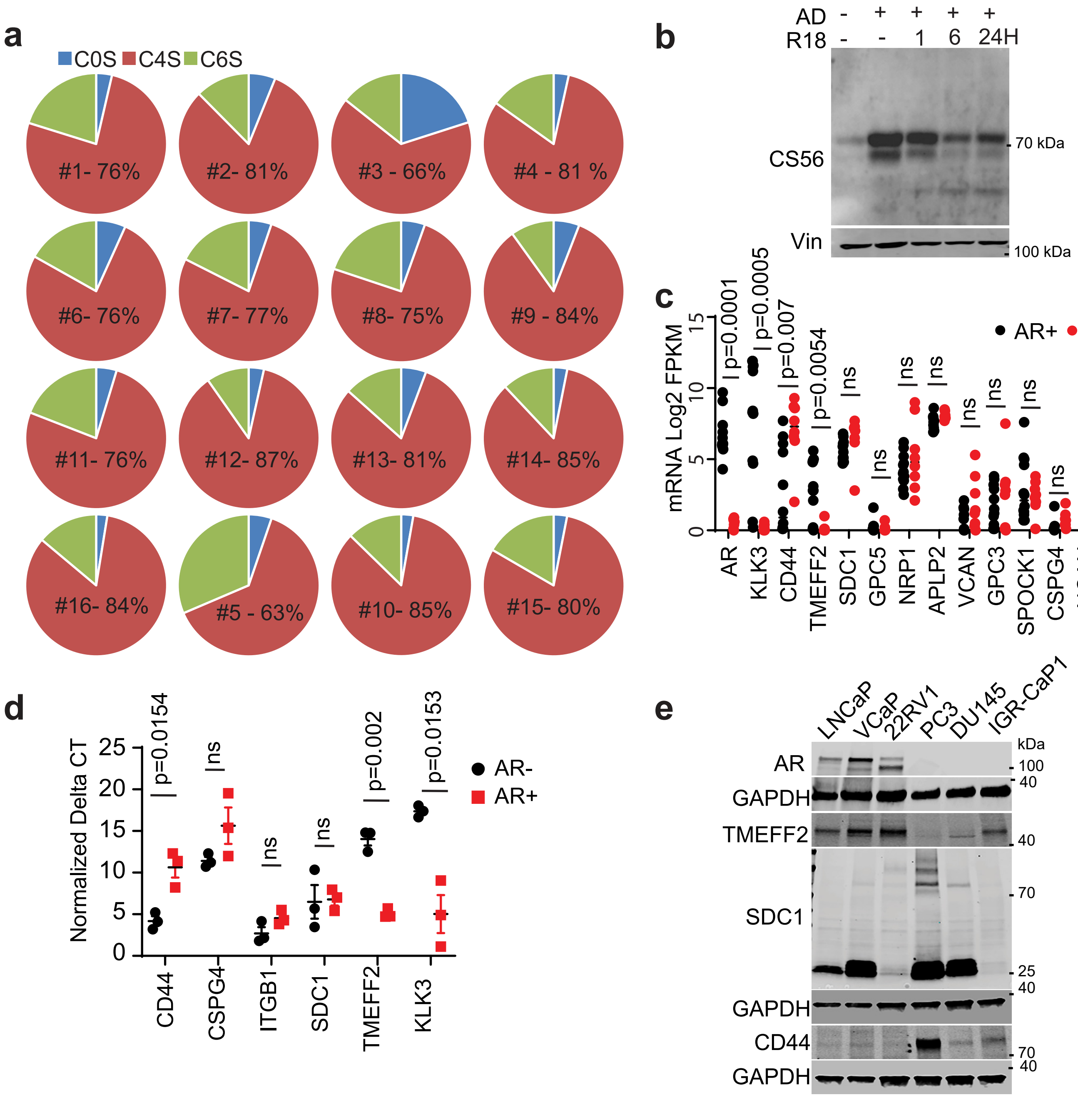

Supplementary Figure 4: Not all CSPG expression is androgen induced

(a) C4S percentage of the total moles of CS disaccharides (C0S+C4S+C6S). CS was digested from sixteen paraffin fixed PC patients' samples using CHase and analyzed by LC-MS. (b) CS56 western blot staining on LNCaP cells after androgen deprivation (AD) and R1881 (10nM) addition at different time points; representative of 3 independent experiments. (c) Gene expression of CSPGs in AR+(22RV1, DuCaP, LAPC4, LNCaP_C4, LNCaP_C42, LNCaP, LNCaP_RF, MDAPCa2b, PC346C, VCAP, CWRR1, LNCaP_19) and AR- (DU145, PC3M, NCIHH60, MDAPCa1, IGRPCaP1, HH870, CaHPV10, PC3) cell lines from Smith et al. PMID: 33303959; two tailed t-test. (d) qRT-PCR delta CT of AR+ (LNCaP, VCaP, 22RV1) and AR- (DU145, PC3, IGR-Cap1) cell lines. Data are presented as mean ± SEM; two tailed t-test. Representative data of n=3 independent experiments. (e) Protein expression by western blot of selected CSPGs in prostate cancer cell lines. Representative data of n=3 independent experiments. ns, non significant. Source data are provided as a Source Data file.

Supplementary Figure 5

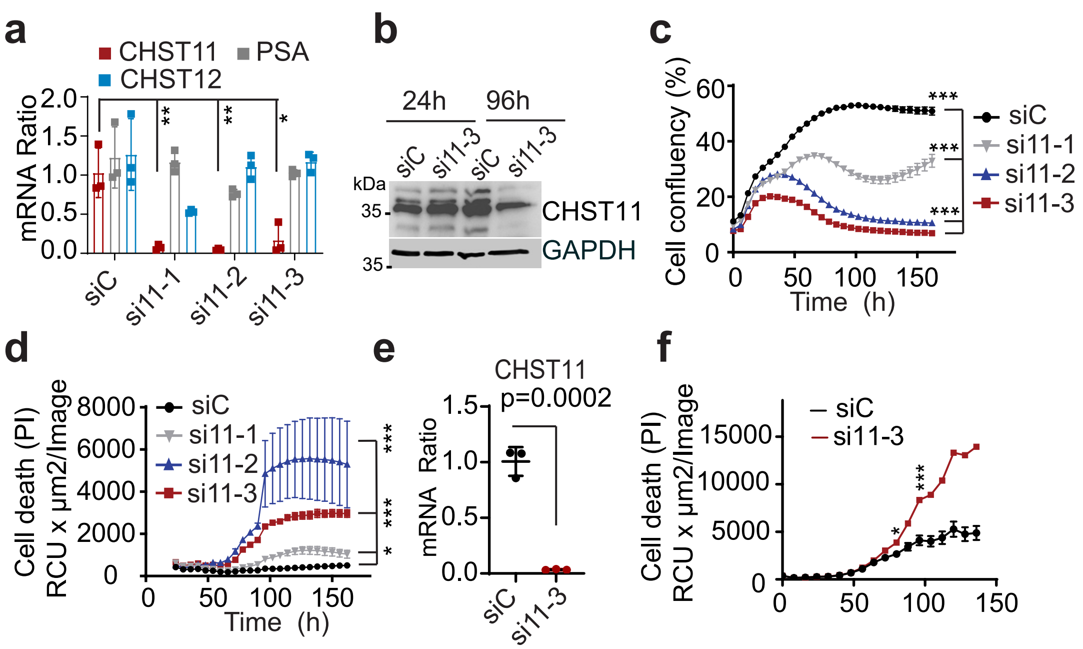

Supplementary Figure 5: CHST11 downregulation induces cell death in PC cell lines
(a) qPCR mRNA expression levels of CHST11 relative to control 48 hours after CHST11 downregulation in LNCaP cells using 3 different si-CHST11 sequences. siC p=0.006; si11-1 p=0.0057; si11-3 p=0.016 by two tailed t-test. CHST12 and PSA were used as control. Data are shown as mean ±SD. n= 3 biologically independent samples. (b) Protein expression of CHST11 in LNCaP cells after 48 and 96 hours of CHST11 downregulation using si11-3. (c) Growth (IncuCyte) of LNCaP cells treated as in (a) Data are shown as mean ± SEM, n=8 biologically independent samples. siC vs si11-1/2/3 p<0.0001 at 24h by two-way ANOVA followed by Dunnett's correction (d) Cell death measurement of (c) using propidium iodide (red staining; Incucyte). Data are shown as mean ± SEM, n=8 biologically independent samples. siC vs si11-2/3 p<0.0001, siC vs si11-1 p<0.05 at 78 h by two-way ANOVA followed by Dunnett's correction. (e) qPCR mRNA level of CHST11 in PC3 cells 48h after CHST11 downregulation using si11-3. Data are shown as mean ± SD. n=3 biologically independent samples. Two tailed t-test. (f) Cell death (IncuCyte) of PC3 cells after CHST11 downregulation using propidium iodide. Data are shown as mean ± SEM, n=6 biologically independent samples. At 72h p=0.027, at 96h p=0.0004 by two tailed t-test. *p<0.05; **p<0.01; ***p<0.001; ****p<0.0001. Source data are provided as a Source Data file.

Supplementary Figure 6

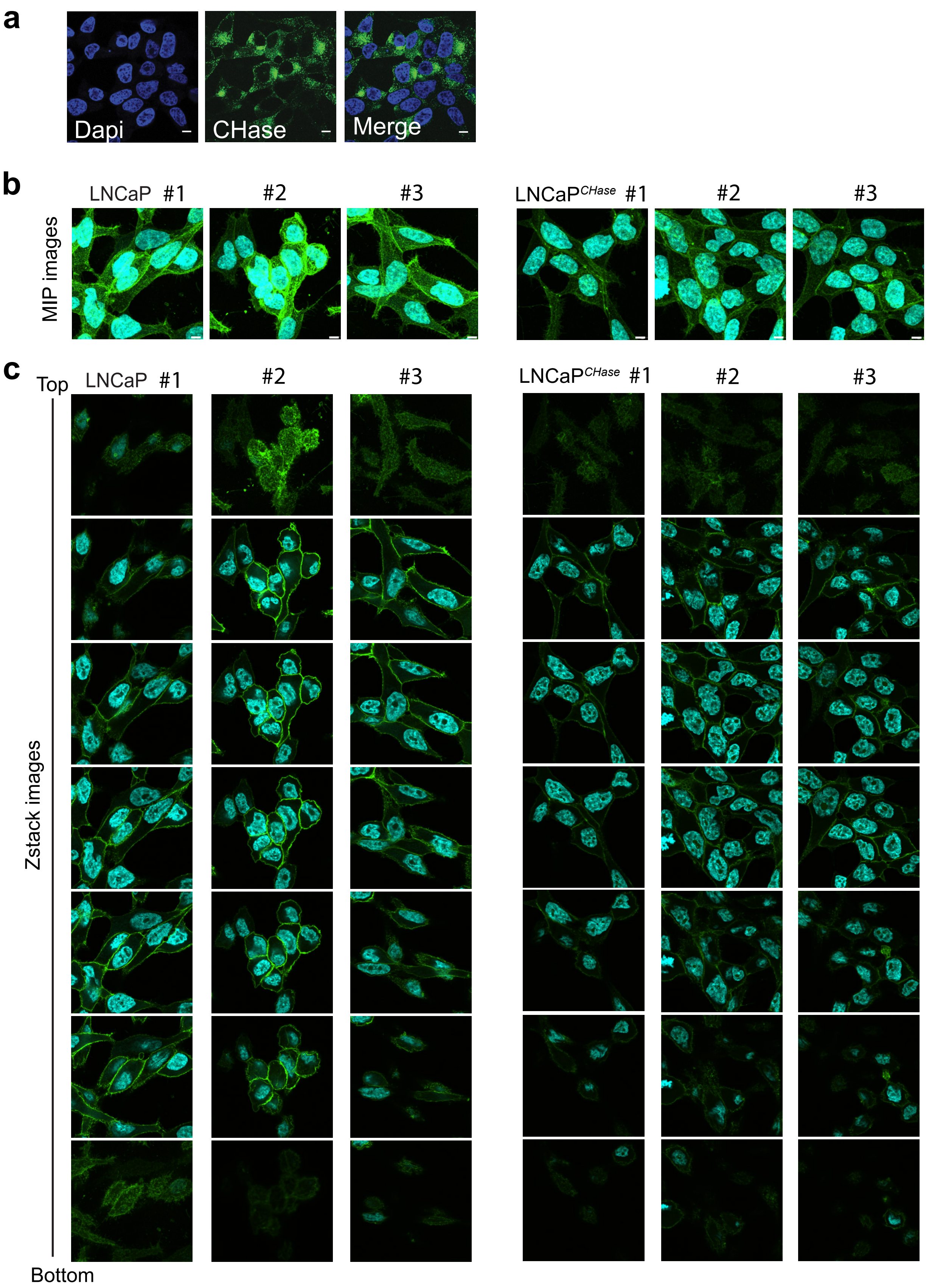

Supplementary Figure 6: LNCaPCHase cells desplay a reduced GalNAz signal

(a) Ectopic expression of CHase (Green) in LNCAPCHase cell line by confocal microscopy. Blue represent DAPI staining. Scale bar is equal to 10μm. (b) Levels of GalNAc in the glycocalyx of LNCaP and LNCaPCHase cells by immunofluorescence using GalNAz. Three representative examples of Maximin Intensity Projection (MIP) photos of LNCaP and LNCaPCHase used for quantification of GalNAz-glycocalyx (green). Hoechst staining (blue) was used to identify the nucleus. Scale bar is 5μm. (c) Z-stack images used to produce MIP figures in (b).

Supplementary Figure 7

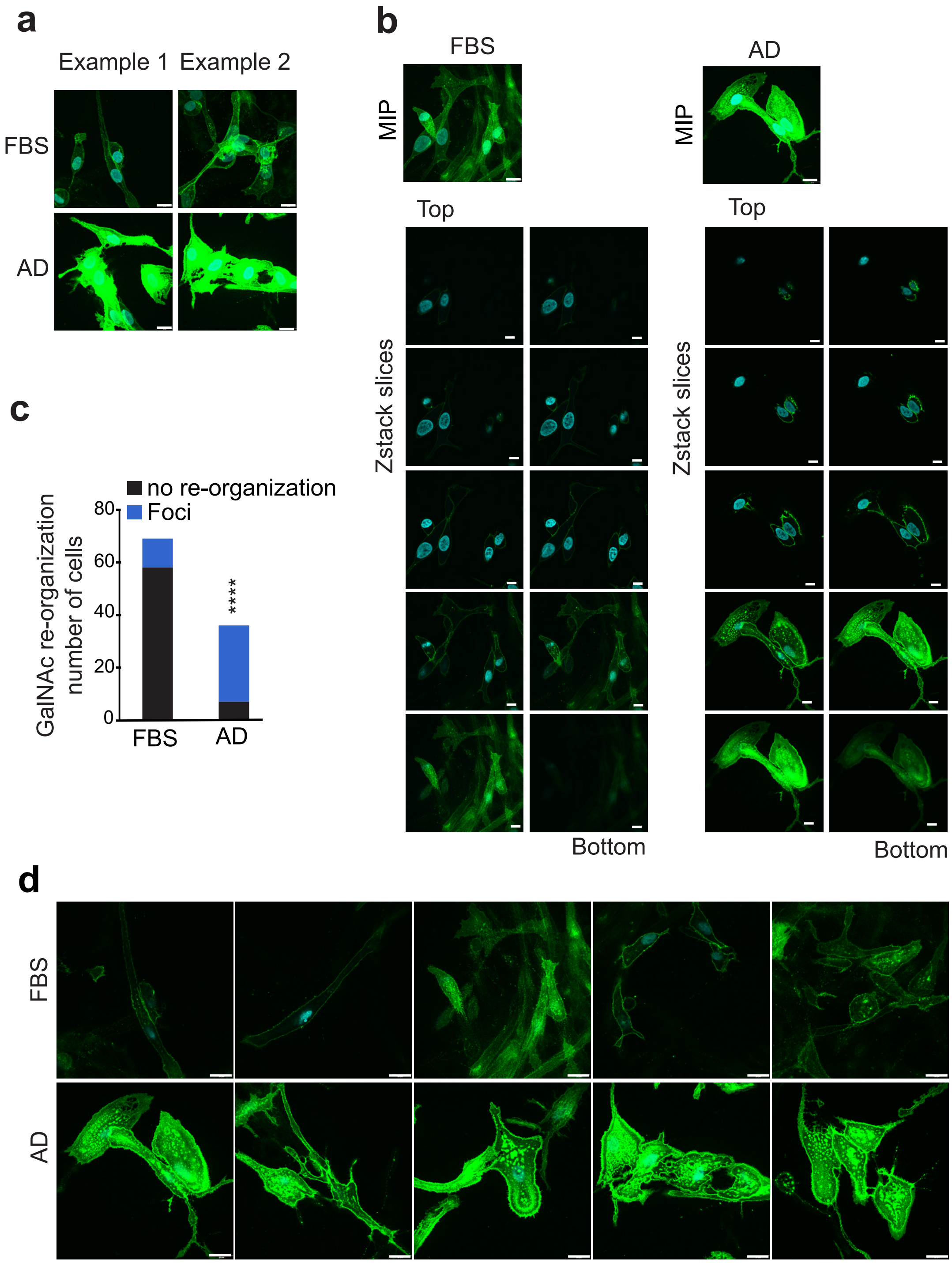

Supplementary Figure 7: Androgen deprivation increases expression of GalNAc-containing glycocalyx

(a) Examples of MIP photos used for quantification in Fig. 5d. Region of interest were drawn around cells and the average pixel intensity was measured using ImageJ. Scale bar = 20um. (b) Z-stack images of Fig.5d. Scale bar = 20μm. (c) Quantification of cells with foci or no reorganization in eleven images for each condition. Same data as Fig. 5f. p<0.0001 by Chi-squared test. (d) Examples of slice images used to quantify GalNAc re-organization in Fig. 5f. Images were acquired at the bottom of the cells. Scale bar = 20μm.

Supplementary Figure 8

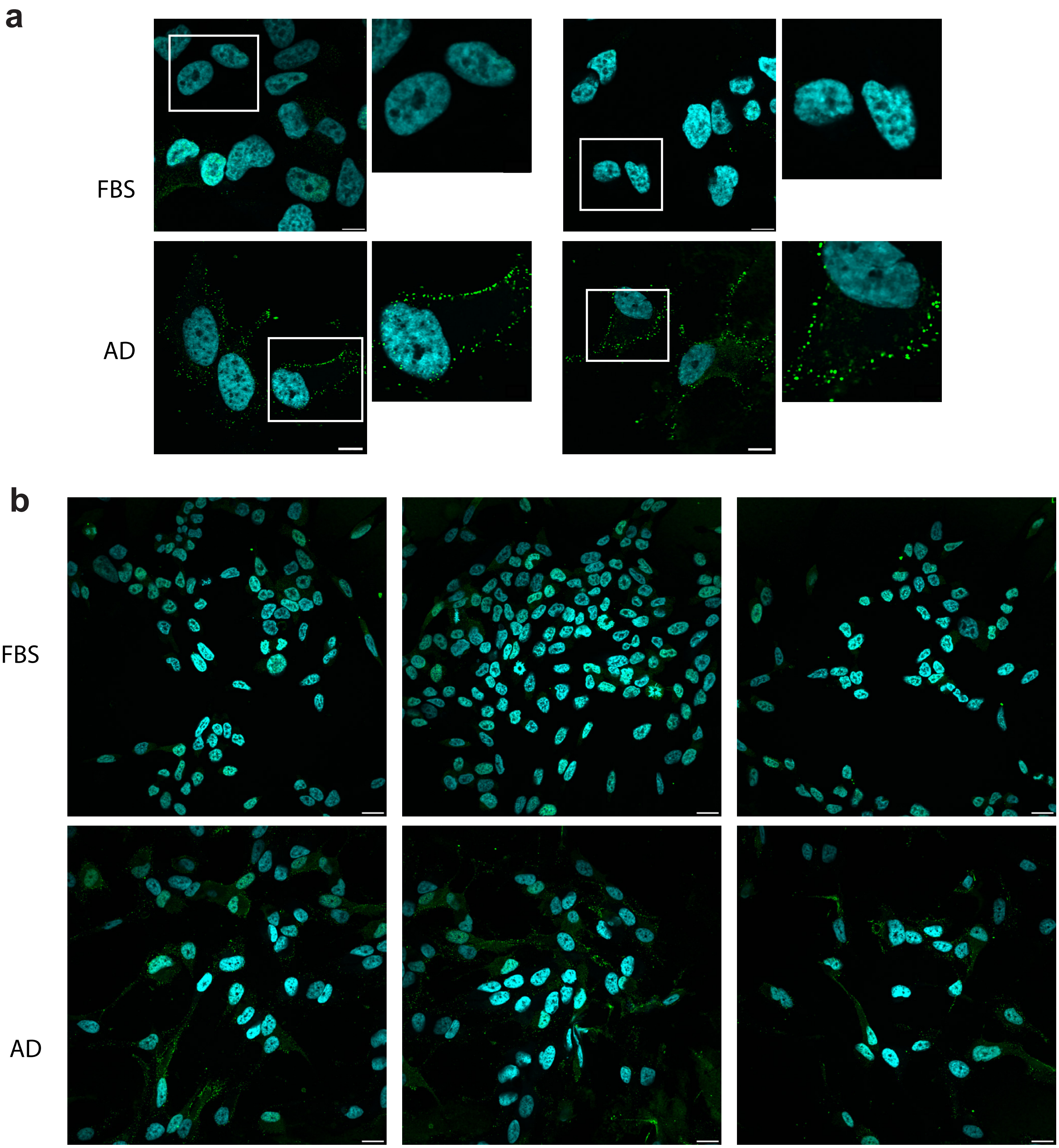

Supplementary Figure 8: Androgen deprivation increases real time binding of VAR2 lectin
(a) rVAR2 staining on LNCaP live cells in FBS or 10 days of AD. rVAR2 was added into live cells for 10 minutes prior to fixation. Hoechst staining (blue) was used to identify the nucleus. Scale bar is 20µm. (b) Representative images of full view of 60x magnifications of (a). Representative data of 3 independent experiments.

Supplementary Figure 9

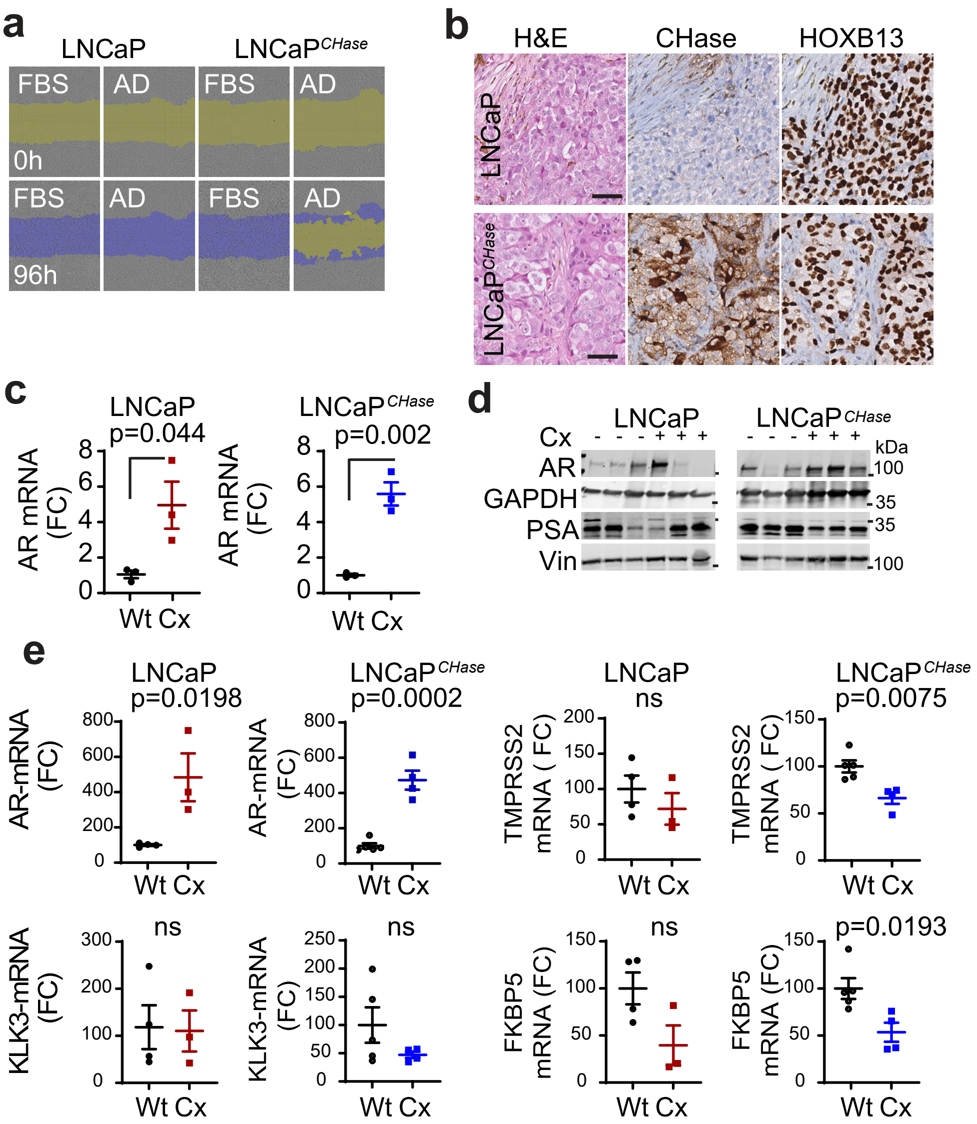

Supplementary Figure 9: Chondroitinase effect on cancer cells

(a) Representative images of Fig. 5h. Wound healing closure measurements of LNCaP and LNCaPCHase cells in FBS or AD conditions, yellow show original scratch wound limits. Blue shows closed wound margins. (b) Immunohistochemistry analysis on LNCaP and LNCaP-CHase at experimental endpoint. HOXB13 was used as a marker of human PC cells. Scale bar represents 50µm. Representative imageS of 5 independent tumors (c) qPCR mRNA expression levels of AR in tumors (n=3 biologically independent tumor samples) from Fig.5k and 5l. Data are shown as mean of fold change (FC) compared to control ± SEM ; two tailed t-test. Wild type, Wt; castrated, Cx. (d) AR and PSA expression levels in Fig. 5k and 5l tumors determined by western blotting. (e) mRNA levels of AR and AR-regulated genes in LNCaP (Wt,n=4; Cx, n= 3) and LNCaPCHase (Wt, n=5; Cx n=4) tumors and determined by RNA-Seq. data represented as mean fold change (FC) of normalized counts ± SEM ; two tailed t-test. ns; non significant.

Supplementary Table 1

Antibody	Source	Identifier	Application	Dilution
Rabbit anti-AR	Cell Signaling	RRID:AB_10691711; Cat#5153	W.B; IP	1:2000; 1:100
Mouse anti-chABC	Novus Biologicals	RRID:AB_11016010; Cat#NBP1-96141	W.B; IF,IHC	1:500; 1:100; 1:100
Mouse anti-CHST11	Millipore Sigma	RRID:AB_1840705; Cat# WH0050515M1	W.B	1:500
Rabbit anti-PSA	Cell Signaling	RRID:AB_2797609; Cat#C5365	W.B	1:1000
Rabbit anti-GAPDH	Cell Signaling	Cat# 5174S	W.B	1:1000
Mouse anti-Vinculin	Abcam	Cat# ab130007	W.B	1:1000
Mouse anti-β-Actin	Sigma	Cat# A2228	W.B	1:1000
Mouse anti-V5	Sigma	Cat# R96025	W.B; FACS; IF	1:1000; 1:200; 1:100
Rabbit anti-HOXB13	Cell Signaling	Cat# 90944S	IHC	1:500
Rabbit IgG	Diagenode	Cat# C15410206	IP	1:1000
Donkey anti-mouse 680/800	LI-COR	RRID:AB_10706167	W.B	1:5000
		RRID:AB_2715510	W.B	1:5000
Donkey anti-rabbit 680/800	LI-COR	RRID:AB_10715072	W.B	1:5000
		RRID:AB_2716622	W.B	1:5000
Donkey anti-mouse 488/594	Thermo Fisher	RRID:AB_141607	W.B	1:5000
		RRID:AB_2535789	W.B	1:5000
Donkey anti-rabbit 488/594	Thermo Fisher	RRID:AB_2535792	W.B	1:5000
		RRID:AB_141637	W.B	1:5000
Rabbit anti Syndecan-1	Cell Signaling	Cat#12922s	W.B	1:1000
Mouse anti TMEFF2	GeneTex	Cat#GTX50037	W.B	1:1000
Mouse anti CD44	Novus Biologicals	Cat# BBA10	W.B	1:250
Rabbit anti-CHST13	Abcam	Cat#155957	W.B	1:500
Mouse anti-CS-56	Abcam	Cat#11570	W.B	1:500

Supplementary Table 1: Antibodies used in this study

Supplementary Table 2

Gene	Forward Primer Sequence	Reverse Primer Sequence
PSA	AGT GCG AGA AGC ATT CCC AAC	CCA GCA AGA TCA CGC TTT TGT T
CHST11	CTATTTCCAAATCATGCGGAGG	AGGACAGCAGTGTTTGAGAG
GAPDH	ACCCAGAAGACTGTGGATGG	CAGTGACTTCCCGTTCAG
RPL32	CCCCTTGTGAAGCCCAAGA	GACTGGTGCCGGATGAACTT
ChIP PSA	TCTGCCTTTGTCCCCTAGAT	AACCTTCATTCCCCAGGACT
ChIP CHST11-1	CCTGAGATGCCAAACTGGAGA	AGGCACTAACACAGAAGGACA
ChIP CHST11-2	AAACAGGCAACTCACTCATGC	CAATTTCAACTCCCAGGGCAC
ChIP CHST11-3	CCAGGTGAAGAATCAGACTTTGC	ACTGGGGCATCTCCAGTTTG
ChIP FKBP5	CCCCCCTATTTTAATCGGAGTAC	TTTTGAAGAGCACAGAACACCCT
ChIP TMPRSS2	TGGTCCTGGATGATAAAAAAAGTTT	GACATACGCCCCACAACAGA
TMEFF2	GGTGTCAGAAGGATCATGTGC	CGTCACATTCTGCACCAAACTGG
CD44	GGAGCAGCACTTCAGGAGGTTAC	GGAATGTGTCTTGGTCTCTGGTAGC
CSPG4	AGGACGAAGGAACCCTAGAGT	CACAGGCACACTGTTGTGGA
Syndecan-1	ACGGCTATTCCCACGTCTC	TCTGGCAGGACTACAGCCTC
Integrin B1	GAAGGGTTGCCCTCCAGA	GCTTGAGCTTCTCTGCTGTT

Supplementary Table 2: List of primer sequences for ChIP and RT-qPCR
