# Supplementary material for: Reformation of the chondroitin sulfate glycocalyx enables progression of AR-independent prostate cancer
Source: Nat Commun. 2022 Aug 13;13:4760. doi: 10.1038/s41467-022-32530-7 (PMC9376089; doi:10.1038/s41467-022-32530-7)
Supplement: Supplementary file 2 — Reporting Summary [file 41467_2022_32530_MOESM2_ESM.pdf]

nature portfolio

Corresponding author(s): Mads Daugaard; Nader Al-Nakouzi

Last updated by author(s): Jul 11, 2022

Reporting Summary

Nature Portfolio wishes to improve the reproducibility of the work that we publish. This form provides structure for consistency and transparency in reporting. For further information on Nature Portfolio policies, see our Editorial Policies and the Editorial Policy Checklist.

Statistics

For all statistical analyses, confirm that the following items are present in the figure legend, table legend, main text, or Methods section.

n/a	Confirmed	
☐	☒	The exact sample size (*n*) for each experimental group/condition, given as a discrete number and unit of measurement
☐	☒	A statement on whether measurements were taken from distinct samples or whether the same sample was measured repeatedly
☐	☒	The statistical test(s) used AND whether they are one- or two-sided<br>*Only common tests should be described solely by name; describe more complex techniques in the Methods section.*
☒	☐	A description of all covariates tested
☐	☒	A description of any assumptions or corrections, such as tests of normality and adjustment for multiple comparisons
☐	☒	A full description of the statistical parameters including central tendency (e.g. means) or other basic estimates (e.g. regression coefficient) AND variation (e.g. standard deviation) or associated estimates of uncertainty (e.g. confidence intervals)
☐	☒	For null hypothesis testing, the test statistic (e.g. *F*, *t*, *r*) with confidence intervals, effect sizes, degrees of freedom and *P* value noted<br>*Give P values as exact values whenever suitable.*
☒	☐	For Bayesian analysis, information on the choice of priors and Markov chain Monte Carlo settings
☒	☐	For hierarchical and complex designs, identification of the appropriate level for tests and full reporting of outcomes
☐	☒	Estimates of effect sizes (e.g. Cohen's *d*, Pearson's *r*), indicating how they were calculated

Our web collection on statistics for biologists contains articles on many of the points above.

Software and code

Policy information about availability of computer code

Data collection	Living Image 4.7.3 (Perkin elmer) was used for bioluminescence data collection. BD FACSDiva for flow cytometry acquisition. Incucyte Chemotaxis software.
Data analysis	All statistical analysis were performed on Graphpad Prism and Excel. Bioluminescence Live imaging analysis was performed on Living Image 4.7.3 (Perkin elmer). FlowJo (v9) and Cyflogic (1.2.1) for FACS analysis. Carl Zeiss (zen) for confocal microscopy analysis. Incucyte chemotaxis software.

For manuscripts utilizing custom algorithms or software that are central to the research but not yet described in published literature, software must be made available to editors and reviewers. We strongly encourage code deposition in a community repository (e.g. GitHub). See the Nature Portfolio guidelines for submitting code & software for further information.

Data

Policy information about availability of data

All manuscripts must include a data availability statement. This statement should provide the following information, where applicable:

- Accession codes, unique identifiers, or web links for publicly available datasets
- A description of any restrictions on data availability
- For clinical datasets or third party data, please ensure that the statement adheres to our policy

Previously published RNA-seq data that were re-analyzed here are available under accession code https://www.ncbi.nlm.nih.gov/geo/query/acc.cgi?acc=GSE147250; https://www.ncbi.nlm.nih.gov/geo/query/acc.cgi?acc=GSE59986. The Chip-SEQ data reanalyzed here were obtained from Cistrome.com; WashU Epigenome Browser using data from Chen et al., 2015; Chng et al., 2012. Source Data are provided with this paper. All other data supporting the findings of this study are available in the Source Data file and from the corresponding author on reasonable request.

Field-specific reporting

Please select the one below that is the best fit for your research. If you are not sure, read the appropriate sections before making your selection.

☒ Life sciences ☐ Behavioural & social sciences ☐ Ecological, evolutionary & environmental sciences

For a reference copy of the document with all sections, see nature.com/documents/nr-reporting-summary-flat.pdf

Life sciences study design

All studies must disclose on these points even when the disclosure is negative.

Sample size	For Immunofluorescence analysis the number of samples was determined by a power analysis. The sample sizes were chosen based on standard numbers for most biological studies. Experiments were performed at least as biological triplicates. For patients data, all available data were used.
Data exclusions	No samples were excluded from analysis in this study.
Replication	A detailed protocol is generated for each experiment. Experiments were replicated successfully three times when applicable or it is indicated otherwise.
Randomization	Randomization was applied for all in vivo experiments. For in vitro experiments samples allocation was random.
Blinding	Investigators were blinded to allocation during data collection and data analysis when applicable.

Reporting for specific materials, systems and methods

We require information from authors about some types of materials, experimental systems and methods used in many studies. Here, indicate whether each material, system or method listed is relevant to your study. If you are not sure if a list item applies to your research, read the appropriate section before selecting a response.

Materials & experimental systems

n/a	Involved in the study	
☐	☒	Antibodies
☐	☒	Eukaryotic cell lines
☒	☐	Palaeontology and archaeology
☐	☒	Animals and other organisms
☒	☐	Human research participants
☐	☒	Clinical data
☒	☐	Dual use research of concern

Methods

n/a	Involved in the study	
☒	☐	ChIP-seq
☐	☒	Flow cytometry
☒	☐	MRI-based neuroimaging

Antibodies

Antibodies used

All antibodies used in this study, their catalogue number, applications and dilutions are listed in Source Data File table 1.
Rabbit anti-AR Cell Signaling Cat #5153
Mouse anti-chABC 1E10 Novus Biologicals Cat#NBP1-96141
Mouse anti-CHST11 Millipore Sigma Cat#WH0050515M1
Rabbit anti-PSA Cell Signaling Cat#C5365
Rabbit anti-GAPDH Cell Signaling Cat# 5174S
Mouse anti-Vinculin Abcam Cat# ab130007
Mouse anti-β-Actin Sigma Cat# A2228
Mouse anti-V5 Sigma Cat# R96025
Rabbit anti-HOXB13 Cell Signaling Cat# 90944S
Rabbit IgG Diagenode Cat# C15410206
Rabbit anti Syndecan-1 Cell Signaling Cat#12922s
Mouse anti TMEFF2 GeneTex Cat#GTX50037
Mouse anti CD44 Novus Biologicals Cat# BBA10
Donkey anti-mouse 680/800 LI-COR RRID:AB_10706167
RRID:AB_2715510
Donkey anti-rabbit 680/800 LI-COR RRID:AB_10715072/RRID:AB_2716622
Donkey anti-mouse 488/594 Thermo Fisher RRID:AB_141607/RRID:AB_2535789
Donkey anti-rabbit 488/594 Thermo Fisher RRID:AB_2535792/RRID:AB_141637
Rabbit anti-rat IgG ImmunoResearch Laboratories Cat#312-005-045

Validation

CHST11 antibody was validated by the manufacturer and further validated in our lab in LNCaP and PC3 cells using a set of 3 different

Validation

CHST11 siRNAs. The other antibodies were validated by the manufacturer as indicated in the data sheets of the suppliers and they were cited in multiple peer reviewed publications. Please see below for application used in this study and species reactivity
Rabbit anti-AR Cell Signaling; #5153; Application (W.B); Reactivity (Human)
Mouse anti-chABC Novus Biologicals ; #NBP1-96141; Application (W.B); Reactivity (Non-species specific); chABC is exogenous protein. The antibody was validated in the present study by W.B, IF and IHC. Parental cell lines and tumors that are negative for ChABC were used as negative control for specificity validation.
Mouse anti-CHST11 Millipore Sigma #WH0050515M1; Application (W.B); Reactivity (human, rat, mouse)
Rabbit anti-PSA Cell Signaling #C5365; Application (W.B); Reactivity (Human)
Rabbit anti-GAPDH Cell Signaling Cat# 5174S; Application (W.B); Reactivity (human, rat, mouse, monkey)
Mouse anti-Vinculin Abcam Cat# ab130007; Application (W.B); Reactivity (human, rat, mouse, African green monkey)
Mouse anti-β-Actin Sigma Cat# A2228; Application (W.B); Reactivity (Drosophila, Hirudo medicinalis, carp, rabbit, wide range, pig, cat, human, rat, chicken, guinea pig, sheep, mouse, bovine, canine)
Mouse anti-V5 Sigma Cat# R96025; Application (W.B; IF); Reactivity (Tag)
Rabbit anti-HOXB13 Cell Signaling Cat# 90944S; Application (W.B; IHC); Reactivity (human, monkey)
Rabbit anti Syndecan-1 Cell Signaling Cat#12922s; Application (W.B); Reactivity (human)
Mouse anti TMEFF2 GeneTex Cat#GTX50037; Application (W.B); Reactivity (mouse, human)
Mouse anti CD44 Novus Biologicals Cat# BBA10; Application (W.B); Reactivity (human)

Eukaryotic cell lines

Policy information about cell lines

Cell line source(s)

HEK293T,NCIH660, LNCaP, PC3, VCaP, DU145, 22RV1 Cell lines were obtained from ATCC. IGR-CaP1 was obtained from Dr. Anne Chauchereau. 42D and V16D cells were obtained from Dr. Martin E. Gleave lab.

Authentication

LNCaP, PC3 and DU145 Cell line authentication testing was performed (STR profiling). VCaP and IGR-CaP1 were used just after acquired from original source.

Mycoplasma contamination

All cells were tested for mycoplasma regularly and were negative.

Commonly misidentified lines
(See ICLAC register)

No commonly misidentified cell lines are used in this study

Animals and other organisms

Policy information about studies involving animals; ARRIVE guidelines recommended for reporting animal research

Laboratory animals

Athymic Nu/Nu Male mice age 6-8 weeks from Jackson laboratory.

Wild animals

The study did not involve any wild animals.

Field-collected samples

The study did not involve samples collected from the filed.

Ethics oversight

All animal studies were performed in accordance with protocols approved by the Animal Care Committee at the University of British Columbia (A19–0324). Mice were maintained in ventilated cages (4 mice per cage), with constant humidity (25–47%) and temperature (21–22 °C), under a 12 h:12 h light:dark cycle, and had ad libitum access to rodent chow diet and drinking water.

Note that full information on the approval of the study protocol must also be provided in the manuscript.

Clinical data

Policy information about clinical studies
All manuscripts should comply with the ICMJE guidelines for publication of clinical research and a completed CONSORT checklist must be included with all submissions.

Clinical trial registration

Provide the trial registration number from ClinicalTrials.gov or an equivalent agency.

Study protocol

Note where the full trial protocol can be accessed OR if not available, explain why.

Data collection

Describe the settings and locales of data collection, noting the time periods of recruitment and data collection.

Outcomes

Describe how you pre-defined primary and secondary outcome measures and how you assessed these measures.

Flow Cytometry

Plots

Confirm that:

☒ The axis labels state the marker and fluorochrome used (e.g. CD4-FITC).

☒ The axis scales are clearly visible. Include numbers along axes only for bottom left plot of group (a 'group' is an analysis of identical markers).

☐ All plots are contour plots with outliers or pseudocolor plots.

☒ A numerical value for number of cells or percentage (with statistics) is provided.

Methodology

Sample preparation	Cells were grown to 70%–80% confluency in appropriate growth media and harvested in CellStripper. Cells were incubated with rVAR2 protein (200–25 nM) in PBS containing 2% fetal bovine serum (FBS) for 30 min at 4°C and binding was analyzed after a secondary incubation with an anti-V5-FITC antibody.
Instrument	FACSCanto II (BD Biosciences)
Software	BD FACSDiva for acquisition and FlowJo and Cyflogic for analysis
Cell population abundance	10,000 gated events were acquired
Gating strategy	FSC/SSC for single cell population gating was used for live cell gating

☒ Tick this box to confirm that a figure exemplifying the gating strategy is provided in the Supplementary Information.
